# Effects of inhaled cannabis high in Δ9-THC or CBD on the aging brain: A translational MRI and behavioral study

**DOI:** 10.3389/fnagi.2023.1055433

**Published:** 2023-02-01

**Authors:** Aymen H. Sadaka, Justin Canuel, Marcelo Febo, Clare T. Johnson, Heather B. Bradshaw, Richard Ortiz, Federica Ciumo, Praveen Kulkarni, Michael A. Gitcho, Craig F. Ferris

**Affiliations:** ^1^Center for Translational NeuroImaging, Northeastern University, Boston, MA, United States; ^2^Department of Psychiatry and Neuroscience, University of Florida College of Medicine, Gainesville, FL, United States; ^3^Psychological and Brain Sciences, Program in Neuroscience, Indiana University, Bloomington, IN, United States; ^4^Department of Chemistry and Biochemistry, New Mexico State University, Las Cruces, NM, United States; ^5^Department of Biological Sciences, Delaware Center for Neuroscience Research, Delaware State University, Dover, DE, United States; ^6^Departments of Psychology and Pharmaceutical Sciences, Northeastern University, Boston, MA, United States

**Keywords:** connectomics, analgesia, diffusion weighted imaging, voxel-based morphometry, midbrain dopaminergic system

## Abstract

With the recent legalization of inhaled cannabis for medicinal and recreational use, the elderly represents one of the newest, rapidly growing cohorts of cannabis users. To understand the neurobiological effects of cannabis on the aging brain, 19–20 months old mice were divided into three groups exposed to vaporized cannabis containing ~10% Δ9-THC, ~10% CBD, or placebo for 30 min each day. Voxel based morphometry, diffusion weighted imaging, and resting state functional connectivity data were gathered after 28 days of exposure and following a two-week washout period. Tail-flick, open field, and novel object preference tests were conducted to explore analgesic, anxiolytic, and cognitive effects of cannabis, respectively. Vaporized cannabis high in Δ9-THC and CBD achieved blood levels reported in human users. Mice showed antinociceptive effects to chronic Δ9-THC without tolerance while the anxiolytic and cognitive effects of Δ9-THC waned with treatment. CBD had no effect on any of the behavioral measures. Voxel based morphometry showed a decrease in midbrain dopaminergic volume to chronic Δ9-THC followed but an increase after a two-week washout. Fractional anisotropy values were reduced in the same area by chronic Δ9-THC, suggesting a reduction in gray matter volume. Cannabis high in CBD but not THC increased network strength and efficiency, an effect that persisted after washout. These data would indicate chronic use of inhaled cannabis high in Δ9-THC can be an effective analgesic but not for treatment of anxiety or cognitive decline. The dopaminergic midbrain system was sensitive to chronic Δ9-THC but not CBD showing robust plasticity in volume and water diffusivity prior to and following drug cessation an effect possibly related to the abuse liability of Δ9-THC. Chronic inhaled CBD resulted in enhanced global network connectivity that persisted after drug cessation. The behavioral consequences of this sustained change in brain connectivity remain to be determined.

## Introduction

Due to the legalization of marijuana and an aging population, it is not surprising that an estimated half of the individuals over the age of 50 use cannabis recreationally or medicinally (Sexton et al., 2019). The two main cannabinoids in cannabis – Δ9-tetrahydrocannabinol (Δ9-THC) and cannabidiol (CBD) – target the endocannabinoid system differently, with the former having a strong affinity toward CB1 receptors in the brain and the latter engaged with several targets (Russo, 2017). A recent study suggests N-acyl-phosphatidylethanolamines-specific phospholipase D (NAPE-PLD) involved in lipid biosynthesis of endocannabinoids in the ascending reticular activating system is a target for CBD reducing autonomic arousal under emotional and physical stress (Sadaka et al., 2021). CB1 receptors are located in various brain regions including the hippocampus, accumbens, prefrontal cortex, and cerebellum (Herkenham et al., 1991; Tsou et al., 1998; Pickel et al., 2004). Δ9-THC and CBD have consistently demonstrated antinociceptive properties in translational research (Rahn et al., 2007, 2014; Deng et al., 2015). In a recent study Alkislar et al., demonstrated that smoked cannabis high in Δ9-THC increases the time for paw withdrawal in rats presenting with chemotherapy-induced cold allodynia (Alkislar et al., 2021). This is consistent with clinical applications of cannabis for a variety of ailments common among the elderly including chronic pain, muscle spasms, and glaucoma (Ebbert et al., 2018; Yang K. H. et al., 2021). Another ailment associated with aging is cognitive decline. Loss of episodic memory, processing speed, and attention are common with healthy aging (Parikh et al., 2016; Fraundorf et al., 2019). Despite this, an examination of the cognitive impact of cannabis among the elderly has been equivocal.

It is well established that chronic cannabis use has deleterious effects on the younger population (Sagar and Gruber, 2018). Cannabis alters brain morphology and function leading to poorer working memory and greater impulsivity (Crane et al., 2013). Clinical and translational research has associated chronic cannabis exposure with alterations in cerebellar grey matter, resting-state functional connectivity, and hippocampal volumes causing the aforementioned cognitive deficits (Yucel et al., 2008; Ashtari et al., 2011; Batalla et al., 2013; Dahlgren et al., 2016; Blithikioti et al., 2019). In three studies exploring the cognitive effect of cannabis among the elderly yielded equivocal results. Dregan and Gulliford determined that those who used cannabis at least once by the age of 42 have improved cognitive function (Dregan and Gulliford, 2012). Meanwhile, Thayer and colleagues and Burggren et al., found no significant differences (Burggren et al., 2018; Thayer et al., 2019). This discrepancy can be explained by several confounding factors including the Δ9-THC concentration, the mode of ingestion, and pharmacological interactions.

Preclinical studies in rodents offer controlled conditions for translational research on the effects of cannabis in the aged. Many of these studies explored cannabis as a treatment for dementia. Aso and colleagues found that a daily injection of Δ9-THC and CBD for 5 weeks reduces memory impairment in advanced and early stages of dementia in a mouse model of Alzheimer’s disease (Aso et al., 2016). Fishbein-Kaminiestky et al., found that a low dose of Δ9-THC protects mice from cognitive dysfunction associated with brain neuroinflammation (Fishbein-Kaminietsky et al., 2014). Bilkei-Gorzo et al. reversed age-related cognitive decline in 12-and 18-month-old mice by continuous exposure to low dose Δ9-THC for 28 days delivered by osmotic minipump (Bilkei-Gorzo et al., 2017). In a simpler design, Sarne and coworkers gave a single injection of low dose Δ9-THC to female mice 24 months of age and reported enhanced memory and learning across several cognitive tasks that mirrored that in young mice (Sarne et al., 2018). Interestingly, the effect on cognition lasted for 7 weeks. Despite these corroborative studies in mice showing low doses of injected Δ9-THC can enhance memory the mode of exposure does not representative the human experience when using cannabis for recreational or medicinal clinical settings.

Due to the lack of consensus surrounding the effects of cannabis among the elderly in the clinical research and the unrepresentative nature of the aforementioned translational research, the present study used behavioral tests to explore the cognitive, anxiolytic, and antinociceptive effects of inhaled vaporized cannabis, and several MR imaging modalities to characterize changes in brain morphology, microarchitecture and connectivity in old mice chronically exposed to vaporized cannabis high in Δ9-THC or CBD or placebo for 28 days and 2 weeks after cessation. We hypothesized there would be differences in behavior, morphology and function between mice chronically exposed to inhaled Δ9-THC and CBD, but any differences would minimize or disappear following the two-week washout period.

## Methods and materials

### Animal usage

Male (*n* = 4) and female (*n* = 19) c57bl/j6 mice were obtained from Delaware State University (Dover, Delaware United States) from the laboratory of Dr. Gitcho. Animals were between 19 and 20 months of age at the start of the experiment. All mice were housed in groups of four, maintained on a reverse 12:12 h light–dark cycle with lights off at 09:00 h, and allowed access to food and water *ad libitum*. All mice were tested for behavior and imaged under dim red illumination between the hrs of 10:00 and 18:00 to avoid the transitions between the circadian light–dark cycle. All mice were acquired and cared for in accordance with the guidelines published in the Guide for the Care and Use of Laboratory Animals (National Institutes of Health Publications No. 85–23, Revised 1985) and adhered to the National Institutes of Health and the American Association for Laboratory Animal Science guidelines. The protocols used in this study complied with the regulations of the Institutional Animal Care and Use Committee at the Northeastern University and adhere to the ARRIVE guidelines for reporting *in vivo* experiments in animal research (Kilkenny et al., 2010).

### Cannabis exposure

Cannabis high in Δ9-THC (10.3% Δ9-THC and 0.05% CBD), high in CBD (10.4% CBD and 0.36% Δ9-THC), and placebo cannabis purported to have less than 0.01%Δ9-THC and 0.01% CBD were acquired from the National Institute on Drug Abuse (NIH/NIDA, Bethesda, MD) through the Research Triangle Institute (Research Triangle Park, NC). Groups of mice were placed in a 38-L exposure chamber (60 cm × 45 cm × 20 cm), that included a vapor inflow tube and several small air outflow holes. Animals were stratified randomly in each group by age and gender (average age of 19.25 month, with 1–2 males and 6–7 females) to ensure that each group were comparable. Subjects were acclimated to the exposure environment for 2 days prior to exposure to reduce any stress of the novel environment. A Volcano Vaporizer (Storz and Bickel, Tuttlingen, Germany) was used to heat cannabis plant material below the point of complete combustion to vaporize the active ingredient (Δ9-THC or CBD), minimizing the generation of harmful free radicals such as polycyclic aromatic hydrocarbons associated with the combustion of organic plant material. The vaporizer was preheated at approximately 210°C and loaded with 0.450 g of minced cannabis. Tubing was attached from the vaporizer to the exposure chamber and the heating fan was run for a total of 60s, filling the exposure chamber with vaporized cannabis aerosols. After 30 min of passive exposure, mice were removed from the exposure chamber and returned to their cages. This exposure protocol occurred daily for 28 consecutive days, in which no adverse reactions were detected. The mass of minced cannabis was based on a previously published study showing that this approach yielded similar serum Δ9-THC concentrations (130–150 ng/ml) to those reported in human users (Farra et al., 2020).

### Evaluation of cannabinoids in plasma

To ensure cannabinoid levels were consistent with treatment, blood samples were collected from each mouse *via* submandibular vein within 30 mins of exposure. Samples were spun down in heparinized tubes, plasma separated and stored at-80°C. Plasma was sent on dry ice to Dr. Bradshaw at Indiana University for cannabinoid lipid analysis. Δ9-THC, CBD, and their metabolites were analyzed as previously described (Leishman et al., 2018). In brief, plasma was rapidly thawed in a water bath, then 90 μl of plasma was added to 2 ml HPLC-grade methanol, the solution was spiked with 100pM deuterium-labeled Anandamide (d8-AEA, Cayman Chemicals), vortexed for 1 min, then left in the dark on ice for 30 min. The solution was centrifuged for 15 min at 19,000× g at 20°C, and the supernatant added to 7.5 ml HPLC water. This solution was partially purified on C18 solid phase extraction columns (Zorbax) and eluted with 65, 75, and 100% methanol. Cannabinoids are concentrated in the 100% methanol fraction. This fraction was analyzed using a C18 reversed-phase analytical column and HPLC/MS/MS detection and Analyst software as previously described (Leishman et al., 2018).

### Behavior

#### Tail flick

Tail flick was used to assess peripheral nociception. Mice were acclimated to a restrainer that restricts movement while also exposing the tail. The tail was placed on a hot plate set to 50°C The time it took for the mouse to flick its tail was recorded, with a ceiling time of 10 s. Tail flick was conducted within the first hr. of cannabis inhalation on day 28 (D28) of chronic exposure and 2 weeks later after washout. Each measure for the three experimental groups was compared with a one-way ANOVA using GraphPad Prism version 9.1.2 for Windows (GraphPad Software, San Diego, California United States). Since the amount of cannabis was limited to 28 days’ worth, and there were various testing modalities that had to be spread over 3 days, OF testing was set on D26, NOR testing was set on D27, and imaging was set on D28.

#### Open field (OF) test

Open Field testing was used to assess anxiety, exploratory behaviors, and locomotor ability. It is based on the natural tendency of an animal to explore and protect itself using avoidance which translates to a normal animal spending more time in the periphery of the open field along the walls of the arena than in the center (the most anxiogenic area). OF was conducted on D1 and D26 of the 28-day exposure period to assess the anxiolytic effects of cannabis after acute and chronic exposure, respectively. Additionally, OF was conducted 15 days after exposure cessation to determine if there were long-term anxiolytic effects.

Animals were placed in a large black cube-shaped Plexiglas box with no lid that was indirectly dimly illuminated with two 40 W incandescent red-light bulbs and allowed to explore for 20 min. For analysis, the arena was divided into a peripheral zone measuring 8 cm from the edge of the arena walls, and a central zone around 40% of the total surface of the arena. The amount of time spent in the periphery and the total distance were determined using ANY-MAZE tracking software. Each measure for the three experimental groups was compared with a one-way ANOVA using GraphPad Prism.

#### Novel object preference (NOP) test

NOP was conducted on D2 and D27 of the 28-day exposure period to assess the cognitive effects of cannabis after acute and chronic exposure, respectively. Similarly, NOP was conducted 16 days after exposure cessation to determine if there were long-term cognitive effects. NOP consisted of one acclimation day and one test day. During the acclimation phase, animals were placed in the NOP box (same as the OF box) and allowed 20 min to explore.

There were two phases that each mouse had to go through during the test day: the Familiar Phase and the Novel Phase. During the Familiar Phase, each animal was placed back in the NOR box for 5 min. The box contained two identical simple objects (e.g., a white solid prism) in two corners of the box diagonal to one another. During this phase of testing, animals were allowed to examine the objects and then placed back in their home cages for at least 90 min before the ‘Novel Phase.’ During the Novel Phase, animals were again placed back into the NOR box. This time, one object from the Familiar phase was replaced with a novel simple object (e.g., a black bottle). Animals were allowed to examine the objects for 3 min and placed back into their home cage. Each session was video-recorded, and ANY-MAZE tracking software was used to determine the amount of time spent with each object to calculate the investigation ratio (IR).

Investigation ratios (IR = time spent investigating the novel object/time spent investigating both objects) were assessed using single-sample, two-tailed t-tests, and performance was compared to chance (i.e., IR = 0.5). An investigation ratio significantly greater than 0.5 indicates that the mice were spending more time with the novel object. Conversely, a ratio significantly smaller than chance was used as an index of a preference for the familiar object. Analysis was performed with GraphPad Prism.

### Neuroimaging

Imaging sessions were conducted under dim-red illumination using a Bruker Biospec 7.0 T/20-cm USR horizontal magnet (Bruker, Billerica, MA, United States) and a 20-G/cm magnetic field gradient insert (ID = 12 cm) capable of a 120-μs rise time. Radio frequency signals were sent and received with a quadrature volume coil built into the animal restrainer (Ekam Imaging, Boston, MA, United States). The design of the restraining system included a padded head support obviating the need for ear bars helping to reduce animal discomfort while minimizing motion artifact. All mice were imaged while under light 1% isoflurane anesthesia for a maximum of 1 hour. The respiration rate was ca 50–55 breaths/min. At the beginning of each imaging session, a high-resolution anatomical data set was collected for volumetric analysis using RARE (Rapid Acquisition with Relaxation Enhancement) pulse sequence with the following parameters: 35 slices of 0.7 mm thickness; field of view [FOV] 3 cm; 256 × 256; a repetition time [TR] of 3,900 msec; an effective echo time [TE] of 48 msec, and number of excitations [NEX] of 3; acquisition time 6 min 14 s.

#### Voxel based morphometry

A 3D Mouse Brain Atlas^©^ with 139 segmented and annotated brain regions (Ekam Solutions; Boston, MA) was used to calculate brain volumes, and register the standard structural mouse template image onto the high resolution T2-weighted images for each individual subject using a non-linear registration method implemented by Unix based software package Deformable Registration *via* Attribute Matching and Mutual-Saliency Weighting (DRAMMS).^1^ Theatlas (image size 256 × 256 × 63) was then warped from the standard space into the subject image space (image size 256 × 256 × 40) using the deformation obtained from the previous step and the nearest-neighbor interpolation method. In the volumetric analysis, each brain region was therefore segmented, and the volume values extracted for all 139 ROIs, calculated by multiplying unit volume of voxel in mm^3^ by the number of voxels using an in-house MATLAB script (available upon request). To account for different brain sizes all the ROI volumes were normalized by dividing each ROI volume by total brain volume of that subject.

Differences in brain volumes (mm^3^) between 139 areas were compared across each of the three experimental conditions (e.g., placebo, Δ9-THC, CBD) using a Kruskal-Wallace nonparametric multiple comparisons test. Volumetric data prior to and following analysis is provided in Supplementary Tables 1 and 2. It was noted that the midbrain dopaminergic system and its primary efferent connections were sensitive to both chronic cannabis and washout conditions. To normalize the data and correct for large differences in brain volumes between areas (e.g., caudate/putamen 16 mm^3^; accumbens core 1.4 mm^3^) the percentage difference in volume between brain areas was calculated. The percent differences for the placebo condition and those for Δ9-THC were compared with a paired t-test.

#### Diffusion weighted imaging – Quantitative anisotropy

Diffusion weighted imaging (DWI) was acquired with a spin-echo echo-planar-imaging (EPI) pulse sequence having the following parameters: TR/TE = 500/20 ms, eight EPI segments, and 10 non-collinear gradient directions with a single b-value shell at 1000 s/mm^2^ and one image with a B-value of 0 s/mm^2^ (referred to as B0). Geometrical parameters were: 48 coronal slices, each 0.313 mm thick (brain volume) and with in-plane resolution of 0.313 × 0.313 mm^2^(matrix size 96 × 96; FOV 30 mm^2^). Image reconstruction included DWI analysis of the DW-3D-EPI images to produce the maps of fractional anisotropy (FA) and apparent diffusion coefficient (ADC). DWI analysis was implemented with MATLAB and MedINRIA (1.9.0)^2^ software. Because sporadic excessive breathing during DWI acquisition can lead to significant image motion artifacts that are apparent only in the slices sampled when motion occurred, each image (for each slice and each gradient direction) was screened, prior to DWI analysis, for motion artifacts; if found, acquisition points with motion artifacts were eliminated from the analysis.

For statistical comparisons between mice, each brain volume was registered to the mouse atlas allowing voxel-and region-based statistics. All image transformations and statistical analyses were carried out using the in-house MIVA software. For each vole, the B0 image was co-registered with the B0 template (using a 6-parameter rigid-body transformation). The co-registration parameters were then applied to the DWI indexed maps for the different indices of anisotropy. Normalization was performed on the maps since they provided the most detailed and accurate visualization of brain structures and allowed for more accurate normalization. Normalization parameters were then applied to all DWI indexed maps. Normalized indexed maps were smoothed with a 0.3-mm Gaussian kernel. To ensure that the anisotropy values were not affected significantly by the pre-processing steps, the ‘nearest neighbor’ option was used following registration and normalization. Statistical differences in measures of DWI between experimental groups were determined using a nonparametric Mann–Whitney U Test (alpha set at 5%). The formula below was used to account for false discoveries from multiple comparisons.


$$P\left(i\right)\leq \frac{i}{V}\;\frac{q}{c\left(V\right)}$$

P(i) is the value of p based on the t-test analysis. Each of 139 ROIs (i) within the brain containing (V) ROIs was ranked in order of its probability value (see Supplementary Tables C–E). The false-positive filter value q was set to 0.2 and the predetermined c(V) set at unity.

#### Resting state functional connectivity

Preprocessing in this study was accomplished by combining Analysis of Functional NeuroImages (AFNI_17.1.12),^3^ FMRIB Software library (FSL, v5.0.9),^4^ Deformable Registration *via* Attribute Matching and Mutual-Saliency Weighting (DRAMMS 1.4.1),^5^ and MATLAB (Mathworks, Natick, MA, United States). Brain tissue masks for resting-state functional images were manually drawn using 3DSlicer^6^ and applied for skull stripping. Motion outliers (i.e., data corrupted by extensive motion) were detected in the dataset and the corresponding time points were recorded and regressed out in a later step. Functional data were assessed for the presence of motion spikes. Any large motion spikes were identified and removed from the time-course signals. This filtering step was followed by slice timing correction from interleaved slice acquisition order. Head motion corrections (six motion parameters) were carried out using the first volume as a reference image. Normalization was completed by registering functional data to the mouse atlas using affine registration through DRAMMS. After quality assurance, band-pass filtering (0.01 Hz ~ 0.1 Hz) was performed to reduce low-frequency drift effects and high-frequency physiological noise for each subject. The resulting images were further detrended and spatially smoothed (full width at half maximum = 0.8 mm). Finally, regressors comprised of motion outliers, the six motion parameters, the mean white matter, and cerebrospinal fluid time series were fed into general linear models for nuisance regression to remove unwanted effects. The region-to-region functional connectivity method was performed in this study to measure the correlations in spontaneous BOLD fluctuations. Briefly, a network is comprised of nodes and edges; nodes being the brain region of interest (ROI) and edges being the connections between regions. 139 nodes were defined using the ROIs segmented from the mouse atlas. Voxel time series data were averaged in each node based on the residual images using the nuisance regression procedure. Pearson’s correlation coefficients across all pairs of nodes were computed for each subject among all three groups. The R-values (ranging from −1 to 1) are z-transformed using the Fisher’s Z transform to improve normality. 139 × 139 symmetric connectivity matrices were constructed with each entry representing the strength of the edge. Group-level analysis was performed to look at the functional connectivity in all three experimental groups. The resulting Z-score matrices from one-group t-tests were clustered using the K-nearest neighbors clustering method to identify how nodes cluster together and form resting state networks. A Z-score threshold of |Z| = 2.3 was applied to remove spurious or weak node connections. Voxel-wise seed analysis was also applied to study the temporal correlation of seed regions to the whole brain. The average time-course of BOLD signals in the three seeds were acquired and used to obtain the parametric maps of Fisher’s Z transformed correlation coefficients for each voxel for each subject. These maps were used to assess connectivity differences in the different experimental groups.

### Functional connectivity processing

Resting state processing was carried out using software tools in Analysis of Functional NeuroImages (AFNI; Cox, 1996), FMRIB Software Library (FSL; Jenkinson et al., 2002), and Advanced Normalization Tools (ANTs; Klein et al., 2009). Binary masks outlining mouse brain boundaries were generated for anatomical and functional scans in MATLAB using Three-Dimensional Pulsed Coupled Neural Networks (PCNN3D; Chou et al., 2011) and these were manually edited. In AFNI, 3dDespike was used to remove time series spikes and 3dvolreg for image volume alignment. Preprocessed scans were cropped and a high-pass temporal filter (<0.009 Hz) was used (3dTproject) to remove slow variations (temporal drift) in the fMRI signal. Independent component analysis (ICA) decomposition was applied using Multivariate Exploratory Optimized Decomposition into Independent Components (FSL MELODIC version 3.0) to assess noise components in each subjects’ native space prior to spatial smoothing and registration. In most cases all components contained noise-related signal along brain edges, in ventricular voxels, and large vessel regions. These components were suppressed using a soft (‘non-aggressive’) regression approach, as implemented in FMRIB Software Library (FSL 6.0.3) using FSL regfilt (Jenkinson et al., 2002). A low-pass filter (>0.12 Hz) and spatial smoothing (0.4 mm FWHM) was then applied to the fMRI scans.

Preprocessed anatomical and fMRI scans were aligned to a parcellated mouse common coordinate framework (version 3, or CCFv3) template (Lein et al., 2007). Bilateral region of interest (ROI)-based nodes (148 total) were created with the guidance of the annotated CCFv3 parcellation and using tools in ITKSNAP and FSL, similar to our previous work in rats (Pompilus et al., 2020; Yang Z. et al., 2021). In ITKSNAP, the template along with the overlaid parcellation were used to find the left hemisphere voxel coordinates for each of the nodes included in this study, which were distributed as evenly as possibly without overlap across the mouse brain template. The node coordinates were positioned in subregions of all areas of the neocortex, thalamus, hippocampus, striatum and were consistent with the anatomical subdivisions of the parcellation (large structures, such as the hippocampus, striatum, and regions of the motor, and somatosensory cortex were assigned multiple nodes). We created 0.6 mm diameter spheric nodes in the template space (resolution: 0.05mm^3^) centered on the voxel coordinates. The right hemispheric representations of the same nodes were then created to complete left and right representations for each node. For subject-to-atlas registration, fMRI scans are upsampled from a native space 0.312 × 0.312 × 1.2 mm resolution (spatially smoothed at 0.4 mm FWHM) to a down sampled template resolution of 0.1mm^3^.

Anatomical images were linearly registered to the mouse template using FSL linear registration tool (FLIRT; Jenkinson et al., 2002), using a correlation ratio search cost, full 180-degree search terms, 12 degrees of freedom and trilinear interpolation. The linear registration output was then nonlinearly warped to template space using ANTs (antsIntroduction.sh script). Anatomical-to-atlas linear and nonlinear transformation matrices were applied to fMRI scans at a later stage. Brain extraction using a mask (see above) was first applied to fMRI scans and the cropped scans were then aligned to their respective higher resolution anatomical scans. Timeseries functional images were split into 600 individual volumes and the first in the series was linearly aligned to the anatomical scan using FLIRT (same parameters as above, except 6 degrees of freedom was used in this step). ANTs (antsRegistrationSyNQuick.sh script) was used to warp the lower resolution functional images to their structural (using a single stage step deformable b-spline syn with a 26-step b-spline distance). Linear and nonlinear warping matrices for fMRI-to-anatomical alignment were applied to individual scans in the time series, then the merged 4-D functional timeseries were moved to the atlas space using the prior anatomical-to-template transformation matrices.

A total of 148 ROI masks, divided into 74 left and 74 right ROI’s, were included in our analyses. Center voxel coordinates were used for 3D network visualizations in BrainNet viewer in MATLAB (Xia et al., 2013). Signal timeseries were extracted from preprocessed fMRI scans with the assistance of ROI mask overlays. This generated 148 individual ROI text files per subject that contained L2-normalized resting state signals as a vector of 200 data points. The timeseries files were used in cross-correlations and in calculations of Pearson r coefficients for every pairwise combinations of ROIs (1dCorrelate in AFNI). The 1dCorrelate program uses bootstrapping to calculate confidence intervals (CI) to determine the significance of the Pearson correlation (less than 5% chance the CI range includes 0 corresponds to a significant correlation). The resulting number of pairwise correlations was 10,730 per subject (after removing 148 self-correlations). Correlation coefficients were imported to MATLAB and Fisher’s transform applied to ensure a normal distribution of z values prior to analyses.

### Graph theory calculations in mouse functional connectivity networks

Details of network analysis and formal descriptions of graph metrics are published (Pompilus et al., 2020). Briefly, weighted matrices were analyzed with Brain Connectivity Toolbox (Rubinov and Sporns, 2010) and MATLAB. Global graph metrics were calculated for edge density thresholds ranging from 2 to 40% (in steps of 2%). The area under the curve (AUC) was calculated for these multi-threshold curves and used for statistical analyses and data presentation (Fornito et al., 2016). Node-specific network measures were calculated at a 16% threshold. We first compared node strength between the groups. Node strength is the sum of edge weights per node. As previously reported, we used a probabilistic approach for community detection to calculate a modularity statistic (Q), which indexes the rate of intra-group connections versus connections due to chance (Blondel et al., 2008). The procedure starts with a random grouping of nodes and iteratively moving nodes into groups which maximize the value of Q. The final number of modules and node assignments to each group (e.g., community affiliation assignments) was taken as the median of 1,000 iterations of the modularity maximization procedure (Pompilus et al., 2020).

To assess network integration and efficiency, we analyzed the clustering coefficient (CC; an index of the number of connected neighbors of a node; Onnela et al., 2005), characteristic path length (CPL; the lowest average number of edges between node pairs), and the small world coefficient (SWI; which is >1 for efficient small world networks; Bassett and Bullmore, 2006). Global efficiency was determined as the average inverse of CPL. Edges were randomly swapped 10 times to generate randomized graphs, preserving original degree and strength distributions (Maslov and Sneppen, 2002; Rubinov and Sporns, 2010). SWI was then calculated as the ratio of lambda to gamma coefficients, where lambda is the ratio of real to random network CC, and gamma the ratio of real to random network CPL (Humphries and Gurney, 2008).

Functional connectivity networks were visualized in BrainNet viewer (Xia et al., 2013). Center coordinates for each node were based on CCFv3 parcellations, as indicated above. A 3D whole brain surface mesh file of the mouse brain template (in *.byu format) was generated using an image binarization command in FSL (fslmaths; Jenkinson et al., 2002) and mesh construction tools in ITKSNAP (Yushkevich et al., 2006). Several 3D mouse brain connectome maps were generated in which size of nodes (spheres) was scaled according to node degree, node strength, betweenness centrality, or eigenvector centrality. Additional 3D maps were generated with node colors representing the module assignment (e.g., community affiliation vector) and node size weighted by modularity index.

Statistical analyses and data plotting were carried out using GraphPad Prism 9. Data were analyzed using two-way analyses of variance (ANOVA with germ free status and conventionalization stage as 2 factors with 2 levels each) post-hoc tests carried out using a Tukey–Kramer or Sidak’s test or non-parametric Komogorov-Smirnov tests with two-stage step procedure false discovery rate correction (q = 0.05).

## Results

### Evaluation of cannabinoids in plasma

**Figure 1:**
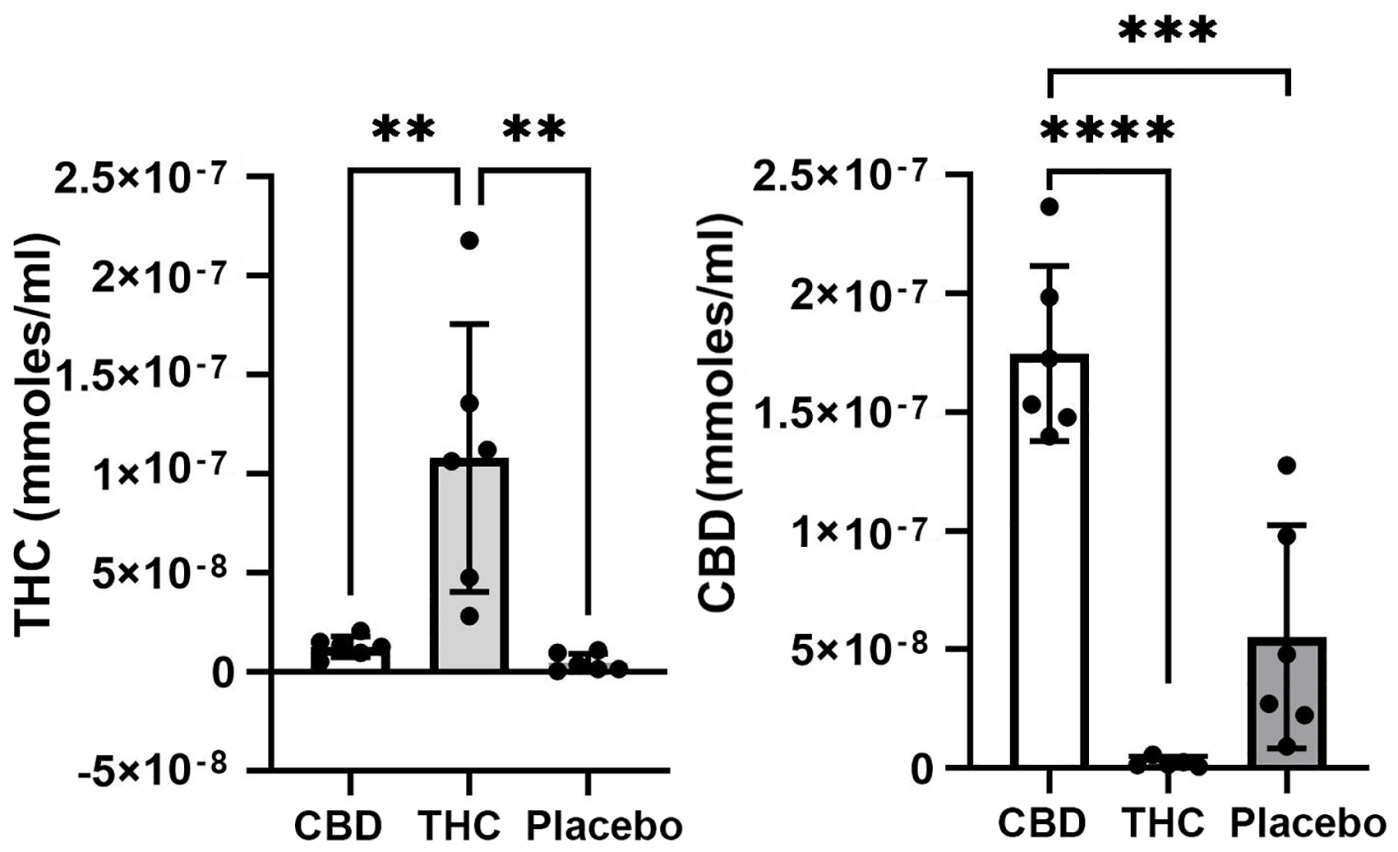
Plasma levels of Δ9-THC and CBD. Shown are scatter plots for blood levels of Δ9-THC and CBD in each of the experimental groups. Note the presence of CBD in mice exposed to placebo. (** *p* < 0.01; *** *p* < 0.001; **** *p* < 0.0001).

Figure 1 shows the plasma concentrations of Δ9-THC and CBD. Comparable levels to previous reports (25–220 ng/ml) were demonstrated in these aged mice. The levels of CBD ranged between 150 and 250 ng/ml and are similar to levels reported in humans smoking cigarettes high in CBD (Hadener et al., 2019; Gelmi et al., 2021). Unexpectedly, placebo had low amounts of CBD that ranged from 10 to 125 ng/ml with 4/6 samples showing levels under 50 ng/ml.

### Behavior

#### Tail flick

**Figure 2:**
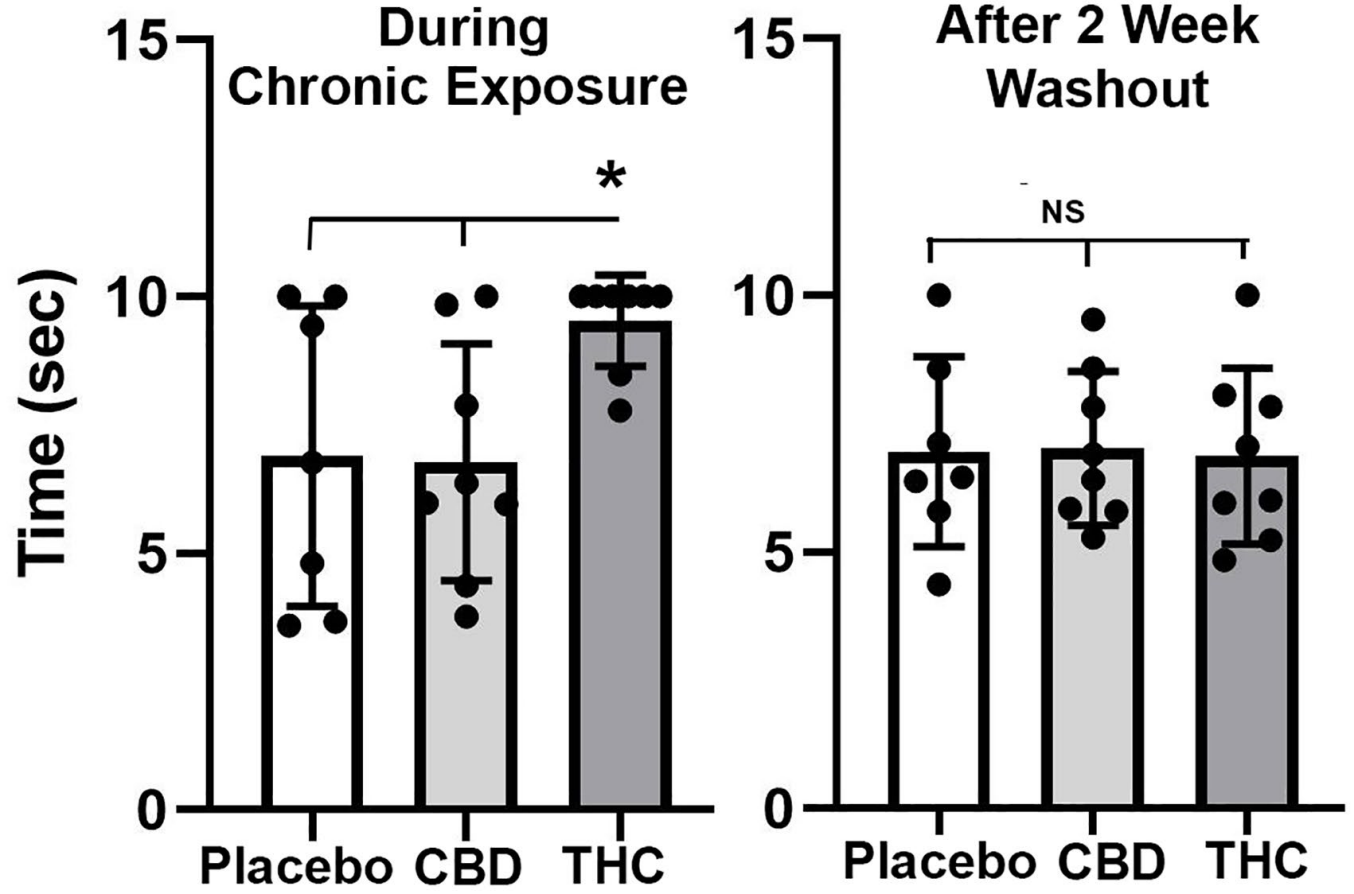
Tail flick. Shown are scatter plots for time to withdraw the tail from the hot water bath for each of the experimental groups during chronic exposure and following a two-week washout. The bar and line represent the mean and SD. (* *p* < 0.05).

Figure 2 shows scatter plots of the amount of time mice spent with their tail on a hotplate after chronic exposure to placebo or vaporized cannabis high in CBD or Δ9-THC and after the washout period. Six out of eight exposed to daily treatments of Δ9-THC for 28 days present with the maximum latency (10s) allowed in the assay. This analgesic effect was significantly greater for Δ9-THC mice than for placebo or CBD groups (*F*_(2,20)_ = 4.00 *p* = 0.038). After a two-week washout without any daily exposure to vaporized cannabis, there were no significant differences between experimental groups as all withdrew their tails in ca 7 s.

#### Open field

**Figure 3:**
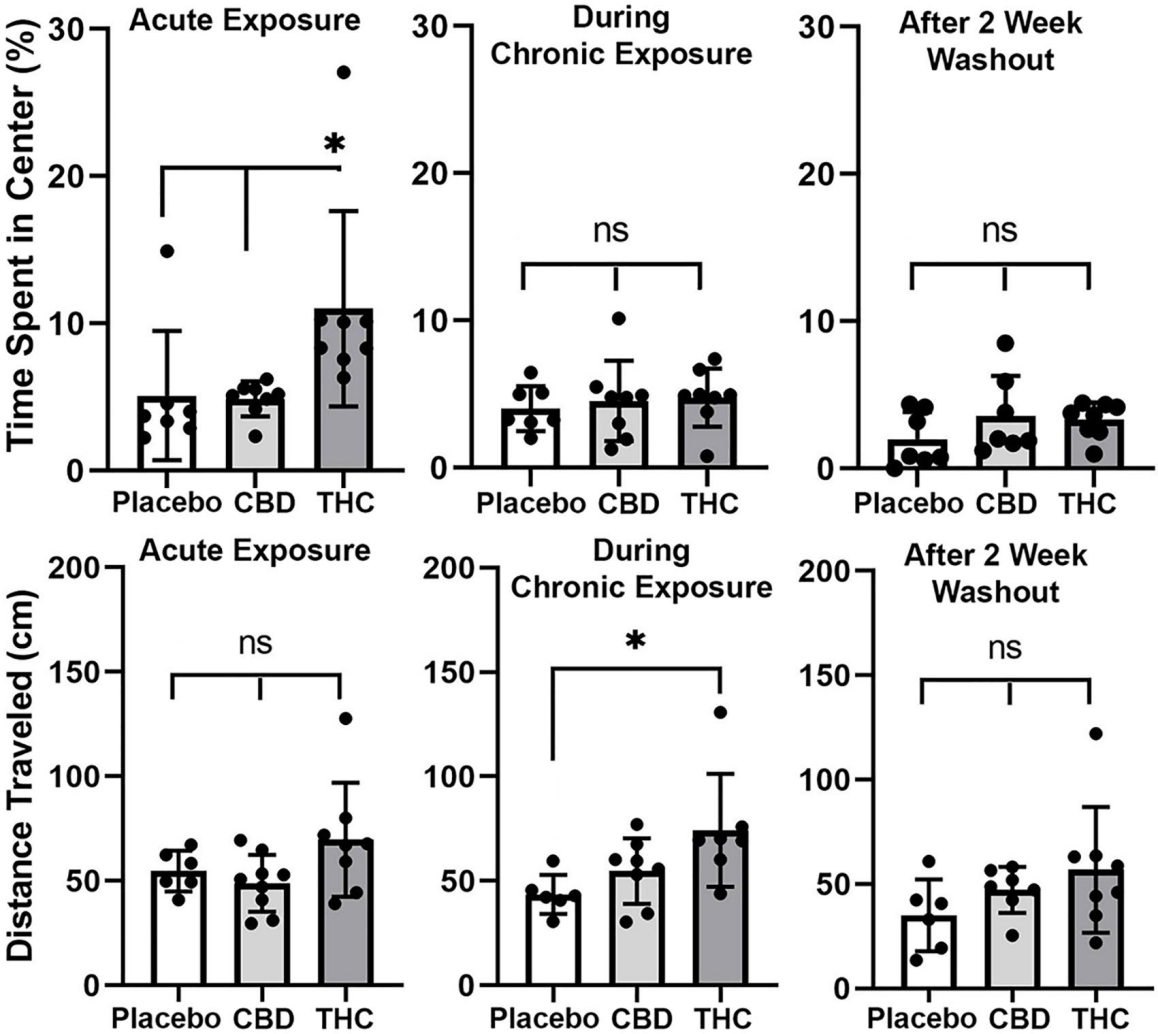
Open field. The scatter plots above show the percentage of time spent in the center of the open field during first exposure (Acute Exposure) to inhaled cannabis high in CBD, Δ9-THC, or placebo and again at D26 of the 28 days of chronic exposure and following a two-week washout. Below is the distance traveled in the open field for each of the experimental conditions at each time point. The bar and line represent the mean and SD, respectively. (* *p* < 0.05).

Shown in Figure 3 are scatter plots of the time spent in the center of the open field arena and the overall distance traveled in the test arena during the 20 min observation period. Mice exposed to vaporized cannabis high in Δ9-THC for the first time (Acute Exposure) spend significantly more time in the center of the test arena than those exposed to placebo and cannabis high in CBD (F_(2,20)_ = 4.381, *p* = 0.023). This would suggest acute exposure to Δ9-THC in old mice has anxiolytic effects. However, this is only true after initial exposure, as it would appear there is tolerance to repeated exposure to Δ9-THC as shown in the middle scatter plots, where there were no significant differences between groups. There were also no significant differences following the two-week washout.

In the bottom panel in Figure 3 are scatter plots for distance traveled. In all cases, across each experimental condition of acute, chronic, and washout of cannabis, mice exposed to Δ9-THC show a greater average distance traveled than with placebo or CBD, but it is only during chronic exposure does this difference achieve significance (*F*_(2, 18)_ = 4.375, *p* = 0.028).

#### Novel object recognition

**Figure 4:**
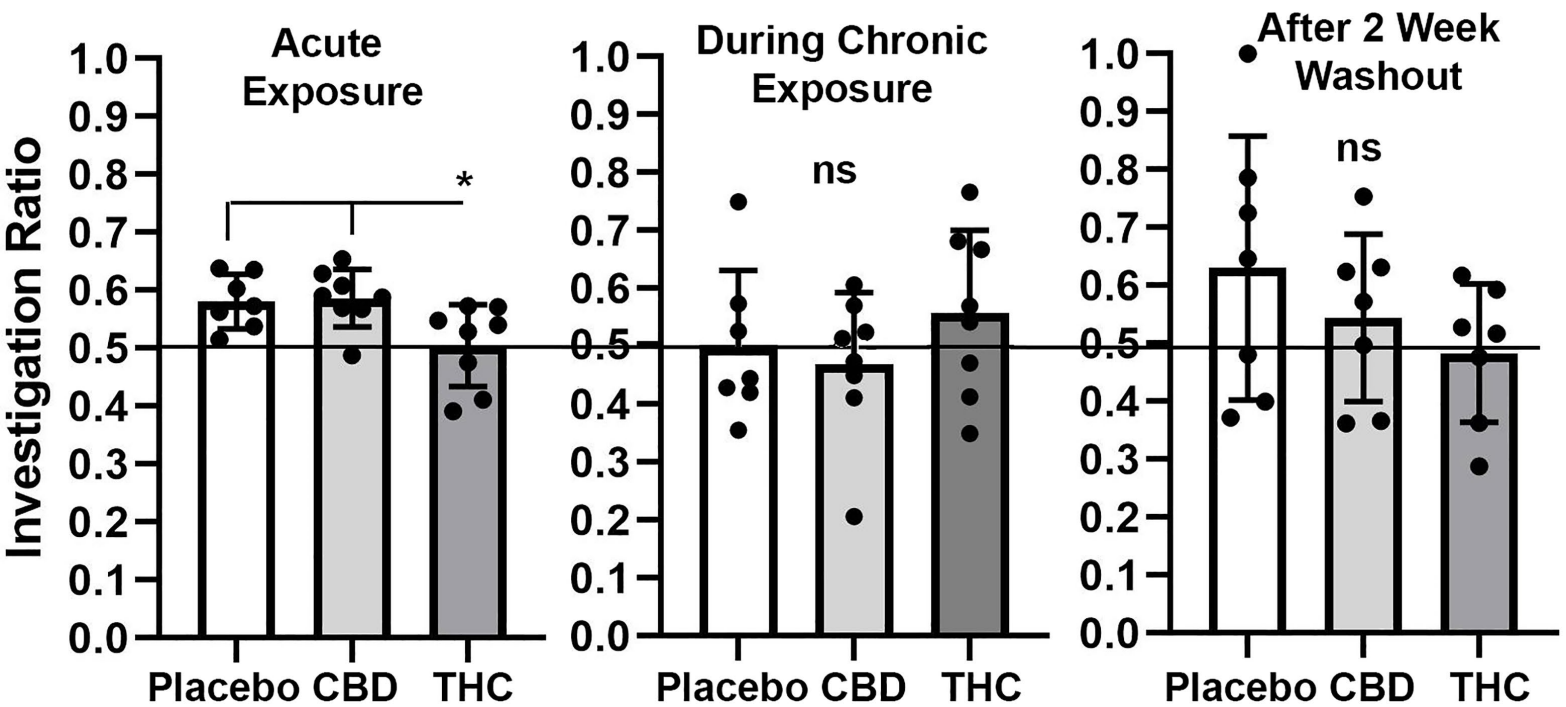
Novel object recognition. Shown are scatter plots of the investigation ratio (time spent investigating the novel object / time spent investigating both objects) using single-sample, two-tailed t-tests, and performance was compared to chance (i.e., IR = 0.5) shown by the horizontal line. measurements were taken during first exposure to inhaled cannabis high in CBD, Δ9-THC, or placebo and again at D27 of the 28 days of chronic exposure and following a two-week washout. The bar and line represent the mean and SD, respectively. (* *p* < 0.05).

Shown in Figure 4 are scatter plots of the investigation ratio or time spent with the novel object as compared to both objects. Mice with acute exposure to vaporized cannabis high in Δ9-THC show significantly less time with the novel object as compared to placebo and CBD groups (F_(2,20)_ = 4.992, *p* = 0.017). Both placebo and CBD were significantly better than chance (*p* = 0.003 and *p* = 0.001, respectively) but not Δ9-THC (*p* = 0.85). Indeed 14 out of 15 mice in the placebo and CBD groups were above the 0.5 line. There were no significant differences between groups during and after chronic exposure to cannabis; although, unlike the first-time mice who were tested in NOR there was an ostensible increase in variance across individuals. None of the mice were significantly greater than chance during chronic exposure and after a two-week washout.

### Neuroimaging

#### Voxel based morphometry

**Figure 5:**
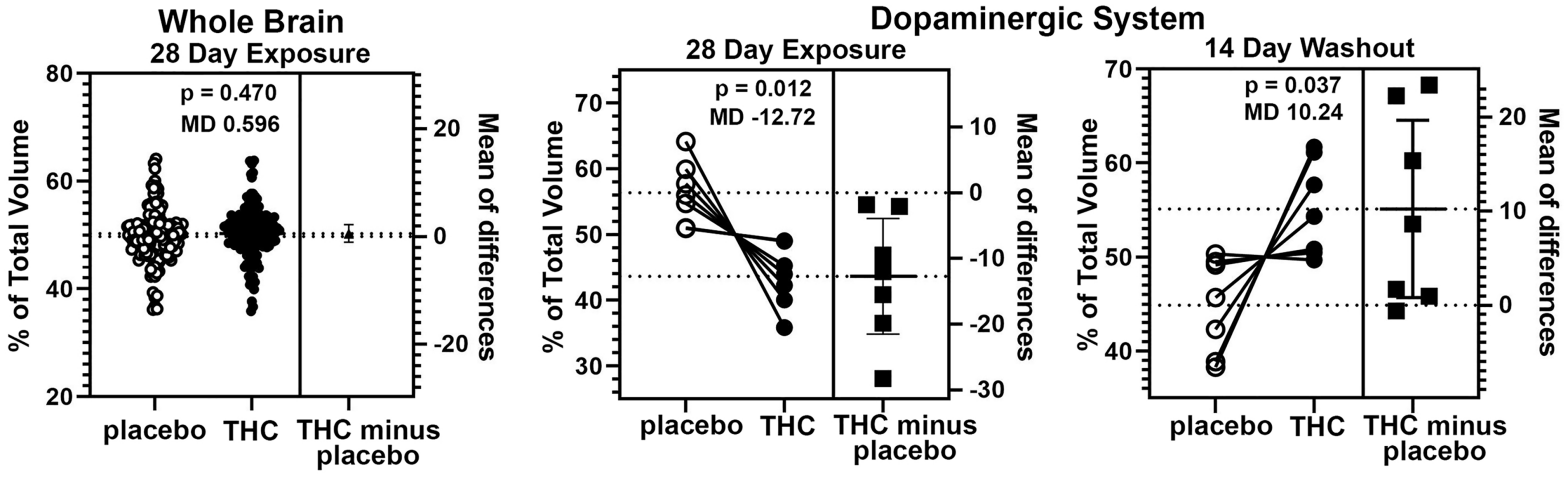
Voxel based morphometry. The scatter plot of the whole brain shows percent of the total volume for 139 brain areas. To normalize the data and correct for large differences in brain volumes between areas the mean percent difference between brain areas was calculated. So each open circle has a matching closed circle and together they equal 100%. All differences between the placebo condition and those for Δ9-THC were compared with a paired t-test. There was no significant difference (*p* = 0.470) with a mean difference (MD) of 0.596. between placebo and Δ9-THC for the whole brain after 28 days of exposure. This is in contrast to the dopaminergic system comprised of the ventral tegmental area, substantia nigra, caudate/putamen, olfactory tubercles, ventral pallidum, accumbens core and shell. In every brain area, the mice exposed to Δ9-THC had percent brain volumes significantly lower (*p* = 0.012) than mice exposed to placebo with a mean difference of-12.17. These same brain areas are significantly larger (*p* = 0.037) with a mean difference of 10.24 than the placebo after the two-week washout of Δ9-THC.

There were few if any significant differences in brain volumes between experimental groups for placebo, CBD, or Δ9-THC during chronic exposure or after the two-week washout (See Supplementary Tables 1 and 2). However, it was noted that mice exposed to chronic Δ9-THC showed reduced volume in brain areas associated with the midbrain dopaminergic system and its major projections. There was a significant (*p* < 0.001) difference in the volume of brain areas comprising the dopaminergic neural circuitry when comparing mice exposed to placebo versus Δ9-THC (Figure 5). The brain areas included in this analysis are the ventral tegmental area (VTA), substantia nigra, caudate/putamen, olfactory tubercles, ventral pallidum, accumbens core and shell. Each of these brain areas are represented by dots in the scatter plot. In every brain area, the mice exposed to Δ9-THC had mean brain volumes significantly lower (*p* = 0.012) than mice exposed to placebo. Interestingly, these same brain areas are significantly larger (*p* = 0.037) than the placebo after the two-week washout of Δ9-THC (Figure 5).

#### Diffusion weighted imaging

**Figure 6:**
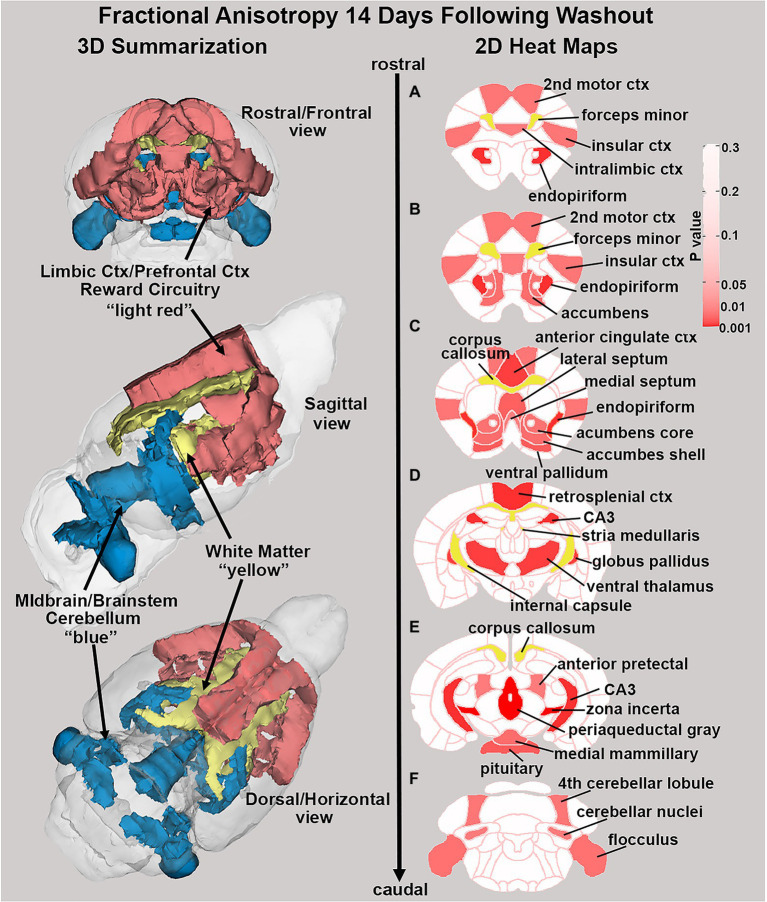
Changes in fractional anisotropy. Shown in the 2D heat maps to the right are brain areas with significantly different FA values following washout of Δ9-THC as compared to placebo. These same areas are reconstructed as 3D volumes to the left. These data were taken from Table 4 and are aligned top (rostral) to bottom (caudal) as 2D axial sections **(A-F)** using the 3D mouse MRI atlas.

There were no significant changes in measures of anisotropy across all brain areas between any experimental groups during 28 days of chronic exposure to cannabis (Supplementary Tables C–E). When comparing chronic exposure to the two-week washout there were still no differences in apparent diffusion coefficient (ADC) as shown in Table 1. However, measures of fractional anisotropy (FA) changed considerably following the washout of cannabis as shown in Table 2. The top panel of Table 2 reports those brain areas during chronic exposure that were significantly different between conditions using a critical value of *p* = 0.05. These eight brain areas, truncated from the 139 areas in the 3D MRI mouse atlas are ranked in order of their significance. Shown are the average measure of FA highlighted in gray and standard deviation for each brain area and their *p*-values and effect size (omega square) in the right columns. The importance of these data has to be considered in the context of the statistics and the use of multiple comparisons as the false discovery rate (FDR) reported to the far right is *p* = 0.015. However, the panel below reports the FA measures across conditions following washout. Note that 29/139 brain areas are significantly different even when considering the FDR of *p* = 0.042. Across the different conditions, placebo, CBD, and Δ9-THC; there is a general trend of decreasing FA values as compared to placebo with the greatest reduction in FA occurring with washout from Δ9-THC. There were 13/139 brains areas that were significantly different between CBD and placebo (Table 3) and 42/139 that were significantly different in FA values between Δ9-THC and placebo as reported in Table 4. The location of many of these brain areas from Table 4 is shown on 2D axial sections of the mouse brain atlas as heat maps denoting their level of significance (Figure 6). Many of the brain areas are in the forebrain (sections A–C) and are associated with the limbic cortex (e.g., insular, infralimbic, and anterior cingulate cortices) and reward circuitry (e.g., accumbens core and shell, ventral pallidum). There is a reduction in FA values in the forceps minor of the corpus callosum the white matter tracts that connect to these forebrain regions. The many brain areas are summarized into brain regions shown in the 3D reconstructions to the left. The volume in light red comprises all areas in sections A–C and the retrosplenial cortex from section D. The area in yellow are all of the white matter tracts that showed a reduction in FA noted in the 2D section. The blue is all the areas from sections D–F.

**Table 1:** Apparent diffusion coefficient during and following chronic cannabis exposure.

	Placebo			CBD			Δ9-THC				
Brain Area	Ave	SD		Ave	SD		Ave	SD	*P* val	Ω Sq	
lateral paragigantocellular n.	1.88	0.33	>	0.85	0.69	<	2.03	0.30	0.004	0.531	
spinal trigeminal n.	1.71	0.27	>	1.38	0.22	<	1.74	0.11	0.007	0.456	FDR
facial n.	1.87	0.32	>	1.44	0.31	>	1.85	0.23	0.017	0.352	*p* = 0.010
paraventricular n. hypo	2.09	0.44	>	1.76	0.42	>	1.51	0.09	0.021	0.328	
parvicellular reticular n.	1.62	0.24	>	1.33	0.15	>	1.55	0.11	0.026	0.300	
pyramidal tracts	1.98	0.36	>	1.59	0.17	>	1.77	0.19	0.034	0.270	
medial dorsal thalamic n.	1.58	0.18	>	1.40	0.13	>	1.35	0.12	0.047	0.233	
Apparent diffusion coefficient following cannabis washout											
	Placebo			CBD			Δ9-THC				
Brain n.	Ave	SD		Ave	SD		Ave	SD	P val	Ω Sq	
cuneate n.	1.77	0.48	<	1.80	0.26	>	1.32	0.13	0.005	0.462	FDR
medial preoptic n.	2.22	0.48	>	1.89	0.20	>	1.72	0.12	0.006	0.445	*p* = 0.008
ambiguus n.	1.98	0.90	<	2.01	0.48	<	2.97	0.38	0.011	0.376	
lateral amygdaloid n.	2.24	0.40	<	2.26	0.58	>	1.56	0.20	0.018	0.326	
basal amygdaloid n.	1.95	0.48	>	1.67	0.27	>	1.50	0.08	0.020	0.311	
central amygdaloid n.	1.81	0.20	>	1.71	0.25	>	1.52	0.09	0.049	0.215	

**Table 2:** Fractional anisotropy during and following chronic cannabis exposure.

	Placebo			CBD			Δ9-THC				
Brain Area	Ave	SD		Ave	SD		Ave	SD	*P* val	Ω Sq	
anterior amygdaloid n.	0.37	0.04	>	0.32	0.01	<	0.39	0.12	0.018	0.343	
endopiriform n.	0.36	0.02	>	0.34	0.02	<	0.40	0.07	0.020	0.330	
infralimbic ctx	0.32	0.06	>	0.26	0.03	<	0.34	0.11	0.023	0.314	FDR
accumbens core	0.43	0.03	>	0.38	0.02	<	0.45	0.08	0.030	0.283	p = 0.015
internal capsule	0.50	0.03	>	0.48	0.02	<	0.52	0.03	0.034	0.269	
prelimbic ctx	0.32	0.10	>	0.25	0.03	<	0.34	0.10	0.036	0.264	
pyramidal tracts	0.46	0.03	>	0.39	0.06	>	0.38	0.08	0.039	0.255	
cuneate n.	0.44	0.09	<	0.52	0.12	>	0.39	0.04	0.047	0.233	
Fractional anisotropy following cannabis washout											
	Placebo			CBD			Δ9-THC				
Brain n.	Ave	SD		Ave	SD		Ave	SD	P val	Ω Sq	
zona incerta	0.48	0.04	>	0.39	0.04	>	0.46	0.04	0.004	0.481	
internal capsule	0.54	0.03	>	0.47	0.05	>	0.47	0.02	0.005	0.472	
globus pallidus	0.48	0.08	>	0.40	0.05	>	0.36	0.01	0.005	0.466	
stria medullaris	0.40	0.07	>	0.38	0.03	>	0.30	0.03	0.007	0.429	
dorsal raphe	0.39	0.06	>	0.33	0.05	>	0.29	0.04	0.008	0.416	
periaqueductal gray	0.40	0.05	>	0.36	0.04	>	0.33	0.02	0.008	0.411	
CA3	0.41	0.07	>	0.37	0.04	>	0.33	0.02	0.008	0.410	
endopiriform n.	0.38	0.05	>	0.33	0.03	>	0.31	0.02	0.009	0.403	
retrosplenial rostral ctx	0.41	0.11	>	0.37	0.07	>	0.28	0.04	0.009	0.399	
forceps minor cc	0.45	0.04	>	0.37	0.07	>	0.35	0.06	0.009	0.399	
anterior cingulate n.	0.38	0.05	>	0.34	0.05	>	0.28	0.05	0.015	0.344	
ventral thalamic n.	0.43	0.04	>	0.36	0.05	>	0.38	0.04	0.016	0.339	
corpus callosum	0.49	0.04	>	0.47	0.06	>	0.40	0.05	0.017	0.329	
extended amygdala	0.48	0.05	>	0.43	0.03	>	0.41	0.04	0.022	0.305	
ventral pallidum	0.42	0.04	>	0.36	0.03	>	0.39	0.03	0.026	0.285	
pituitary	0.43	0.13	<	0.44	0.12	>	0.30	0.06	0.028	0.278	
lateral septal n.	0.44	0.02	>	0.40	0.04	>	0.38	0.03	0.028	0.275	
median raphe n.	0.46	0.09	>	0.41	0.05	>	0.38	0.04	0.030	0.270	
medial mammillary n.	0.39	0.08	<	0.42	0.15	>	0.30	0.02	0.036	0.250	
accumbens shell	0.38	0.05	>	0.33	0.05	>	0.32	0.04	0.039	0.239	
accumbens core	0.41	0.04	>	0.37	0.03	>	0.36	0.03	0.042	0.232	
flocculus cerebellum	0.48	0.12	>	0.43	0.06	>	0.35	0.03	0.042	0.231	
secondary motor ctx	0.36	0.09	>	0.33	0.07	>	0.26	0.03	0.044	0.227	
anterior thalamic n.	0.38	0.03	>	0.34	0.04	>	0.33	0.03	0.045	0.224	
insular rostral ctx	0.39	0.05	>	0.33	0.04	>	0.33	0.04	0.047	0.221	
4th cerebellar lobule	0.47	0.09	>	0.39	0.05	>	0.38	0.03	0.047	0.221	
infralimbic ctx	0.33	0.09	>	0.27	0.06	>	0.23	0.02	0.048	0.219	FDR
cerebellar nuclear n.	0.43	0.08	>	0.38	0.04	>	0.34	0.04	0.048	0.218	*p* = 0.042
anterior pretectal n.	0.39	0.07	>	0.34	0.05	>	0.31	0.02	0.050	0.213	

**Table 3:** Fractional Anisotropy following cannabis CBD washout

Fractional Anisotropy Washout								
	Placebo								CBD			
Brain Area	Ave	SD		Ave	SD	P val	Ω Sq	
zona incerta	0.48	0.04	>	0.39	0.04	0.004	0.597	
ventral thalamic area	0.43	0.04	>	0.36	0.05	0.009	0.472	
ventral pallidum	0.42	0.04	>	0.36	0.03	0.010	0.453	
internal capsule	0.54	0.03	>	0.47	0.05	0.010	0.446	
forceps minor corpus callosum	0.45	0.04	>	0.37	0.07	0.020	0.349	FDR p = 0.017
endopiriform area	0.38	0.05	>	0.33	0.03	0.024	0.325	
insular rostral ctx	0.39	0.05	>	0.33	0.04	0.034	0.277	
insular caudal ctx	0.44	0.08	>	0.35	0.05	0.034	0.275	
accumbens shell	0.38	0.05	>	0.33	0.05	0.035	0.273	
dorsal raphe	0.39	0.06	>	0.33	0.05	0.040	0.252	
reuniens thalamic area	0.41	0.04	>	0.34	0.06	0.046	0.234	
parafascicular thalamic area	0.37	0.09	>	0.28	0.04	0.047	0.232	
parabrachial area	0.45	0.08	>	0.38	0.03	0.047	0.231	

**Table 4:** Fractional anisotropy following cannabis Δ9-THC washout.

	Placebo			Δ9-THC				
BrainArea	Ave	SD		Ave	SD	*P* val	Ω Sq	
globus pallidus	0.48	0.08	>	0.36	0.01	0.002	0.694	
internal capsule	0.54	0.03	>	0.47	0.02	0.003	0.655	
CA3	0.41	0.07	>	0.33	0.02	0.004	0.603	
periaqueductal gray	0.40	0.05	>	0.33	0.02	0.004	0.595	
endopiriform n.	0.38	0.05	>	0.31	0.02	0.005	0.563	
corpus callosum	0.49	0.04	>	0.40	0.05	0.006	0.530	
forceps minor cc	0.45	0.04	>	0.35	0.06	0.006	0.530	
median raphe n.	0.46	0.09	>	0.38	0.04	0.007	0.509	
dorsal raphe	0.39	0.06	>	0.29	0.04	0.007	0.502	
lateral septal n.	0.44	0.02	>	0.38	0.03	0.007	0.502	
anterior cingulate n.	0.38	0.05	>	0.28	0.05	0.007	0.501	
stria medullaris	0.40	0.07	>	0.30	0.03	0.009	0.472	
retrosplenial rostral ctx	0.41	0.11	>	0.28	0.04	0.009	0.471	
infralimbic ctx	0.33	0.09	>	0.23	0.02	0.012	0.422	
medial septal n.	0.41	0.04	>	0.36	0.04	0.014	0.398	
extended amydala	0.48	0.05	>	0.41	0.04	0.015	0.392	
anterior thalamic n.	0.38	0.03	>	0.33	0.03	0.017	0.372	
habenular n.	0.43	0.05	>	0.36	0.05	0.020	0.347	
4th cerebellar lobule	0.47	0.09	>	0.38	0.03	0.021	0.343	
secondary motor ctx	0.36	0.09	>	0.26	0.03	0.024	0.326	
accumbens core	0.41	0.04	>	0.36	0.03	0.024	0.323	
accumbens shell	0.38	0.05	>	0.32	0.04	0.025	0.321	
cerebellar nuclear n.	0.43	0.08	>	0.34	0.04	0.025	0.321	
medial mammillary n.	0.39	0.08	>	0.30	0.02	0.025	0.320	
prelimbic ctx	0.32	0.06	>	0.24	0.05	0.025	0.317	
anterior pretectal n.	0.39	0.07	>	0.31	0.02	0.026	0.313	
mesencephalic reticulum	0.44	0.08	>	0.37	0.02	0.029	0.299	
insular rostral ctx	0.39	0.05	>	0.33	0.04	0.033	0.282	
medial amygdaloid n.	0.46	0.04	>	0.42	0.03	0.033	0.279	
orbital ctx	0.33	0.05	>	0.27	0.03	0.034	0.275	
lateral posterior thalamic n.	0.40	0.07	>	0.33	0.04	0.040	0.254	
parafascicular thalamic n.	0.37	0.09	>	0.28	0.02	0.040	0.252	
ambiguus n.	0.52	0.08	>	0.41	0.07	0.041	0.252	
paraventricular thalamic n.	0.36	0.06	>	0.29	0.04	0.041	0.252	
caudate putamen	0.39	0.09	>	0.30	0.02	0.045	0.236	
primary motor ctx	0.35	0.08	>	0.26	0.03	0.045	0.236	
anterior olfactory n.	0.39	0.04	>	0.35	0.03	0.046	0.236	
posterior hypothalamic n.	0.41	0.09	>	0.32	0.04	0.046	0.234	
medial dorsal thalamic n.	0.37	0.07	>	0.30	0.03	0.047	0.232	
reuniens thalamic n.	0.41	0.04	>	0.35	0.03	0.053	0.216	
pituitary	0.43	0.13	>	0.30	0.06	0.054	0.214	FDR
basal amygdaloid n.	0.44	0.07	>	0.36	0.04	0.055	0.212	*p* = 0.056

#### Connectomics

**Figure 7:**
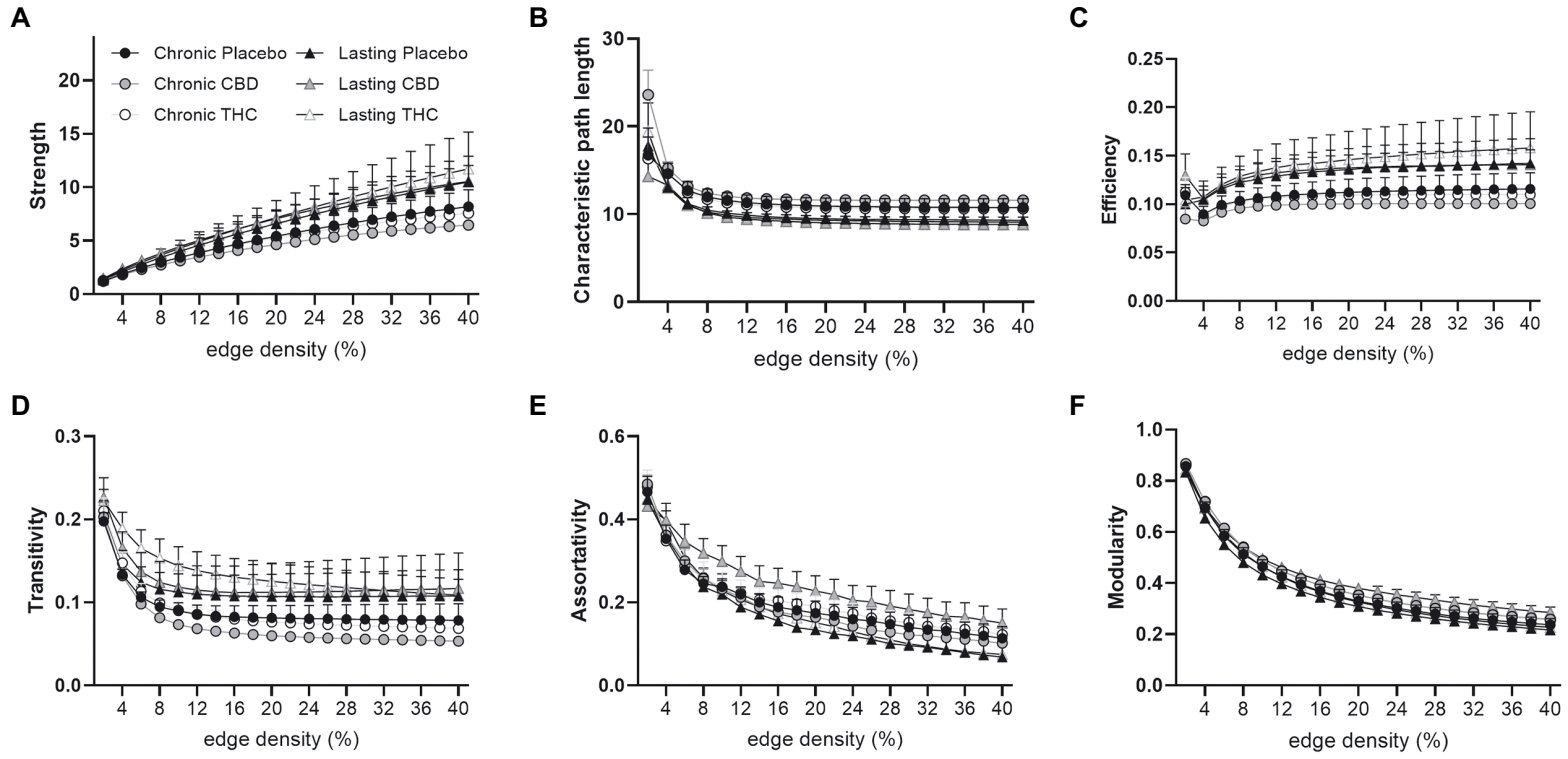
Functional network topology. Functional network topological measures in mice chronically administered vehicle, CBD, or THC 28 days and imaged immediately after (chronic) and after a 2-week washout period (lasting). The network measures shown include **(A)** node strength, **(B)** characteristic path length, **(C)** efficiency, **(D)** transitivity, **(E)** assortativity and **(F)** modularity. All network measures are plotted as a function of edge density threshold (data are shown as mean ± standard error).

**Figure 8:**
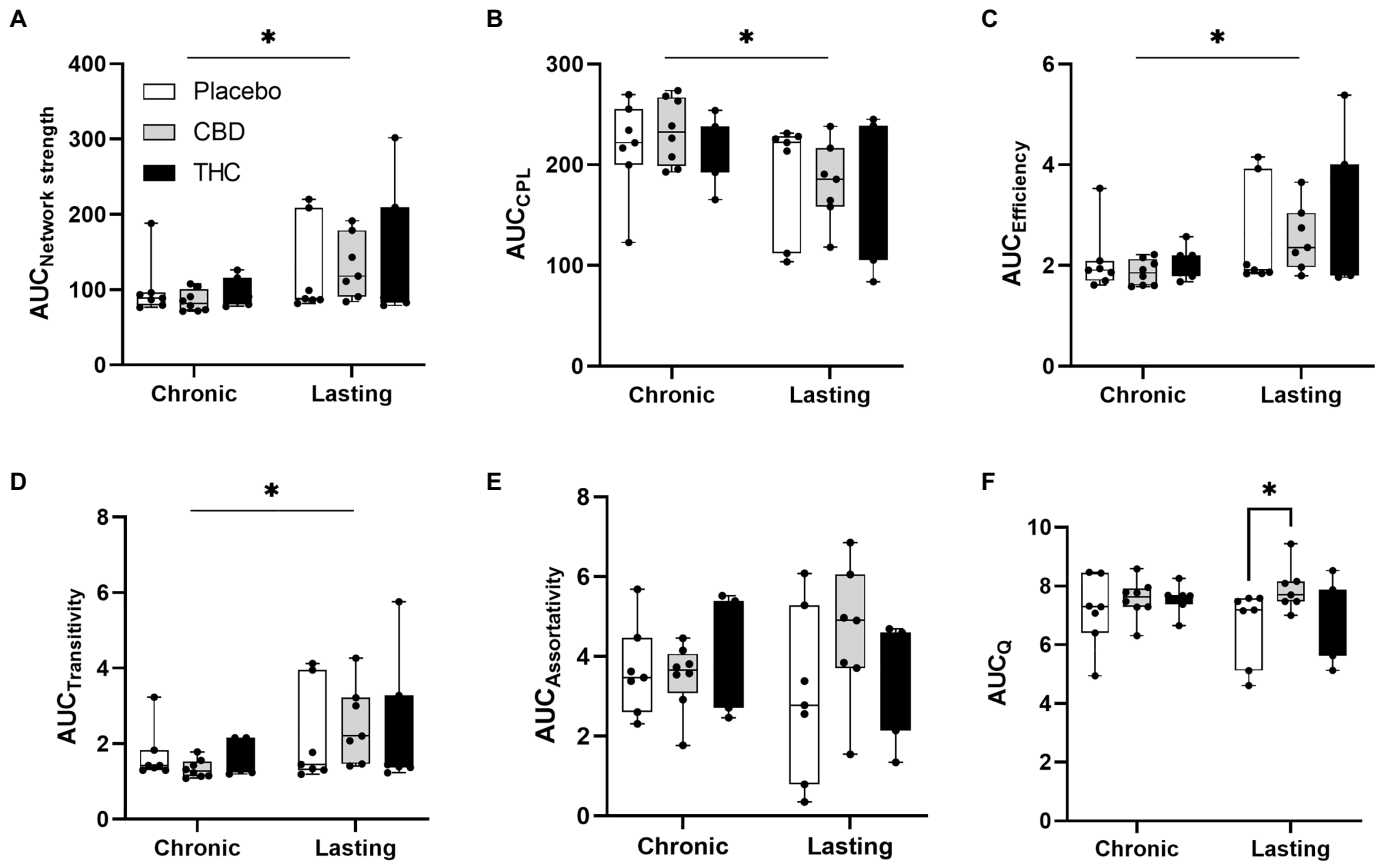
Network modularity. CBD administration results in increased modularity following a 2-week washout period. The network measures shown include **(A)** node strength, **(B)** characteristic path length, **(C)** efficiency, **(D)** transitivity, **(E)** assortativity and **(F)** modularity. Strength, CPL, efficiency, transitivity differed between chronic and lasting imaging sessions (two-way ANOVA main effect of imaging epoch). Data are presented as area under the curve (AUC) values for network measures in Figure 7 calculated for multiple edge density thresholds (2–40%). Individual data points are overlaid on box and whisker plots (mean-max). Statistical analyses done using two factor ANOVA (main effect imaging epoch and drug treatment with Dunnett’s multiple comparison *post-hoc* test *p < 0.05). Abbreviations: CPL, characteristic path length; Q, modularity index.

**Figure 9:**
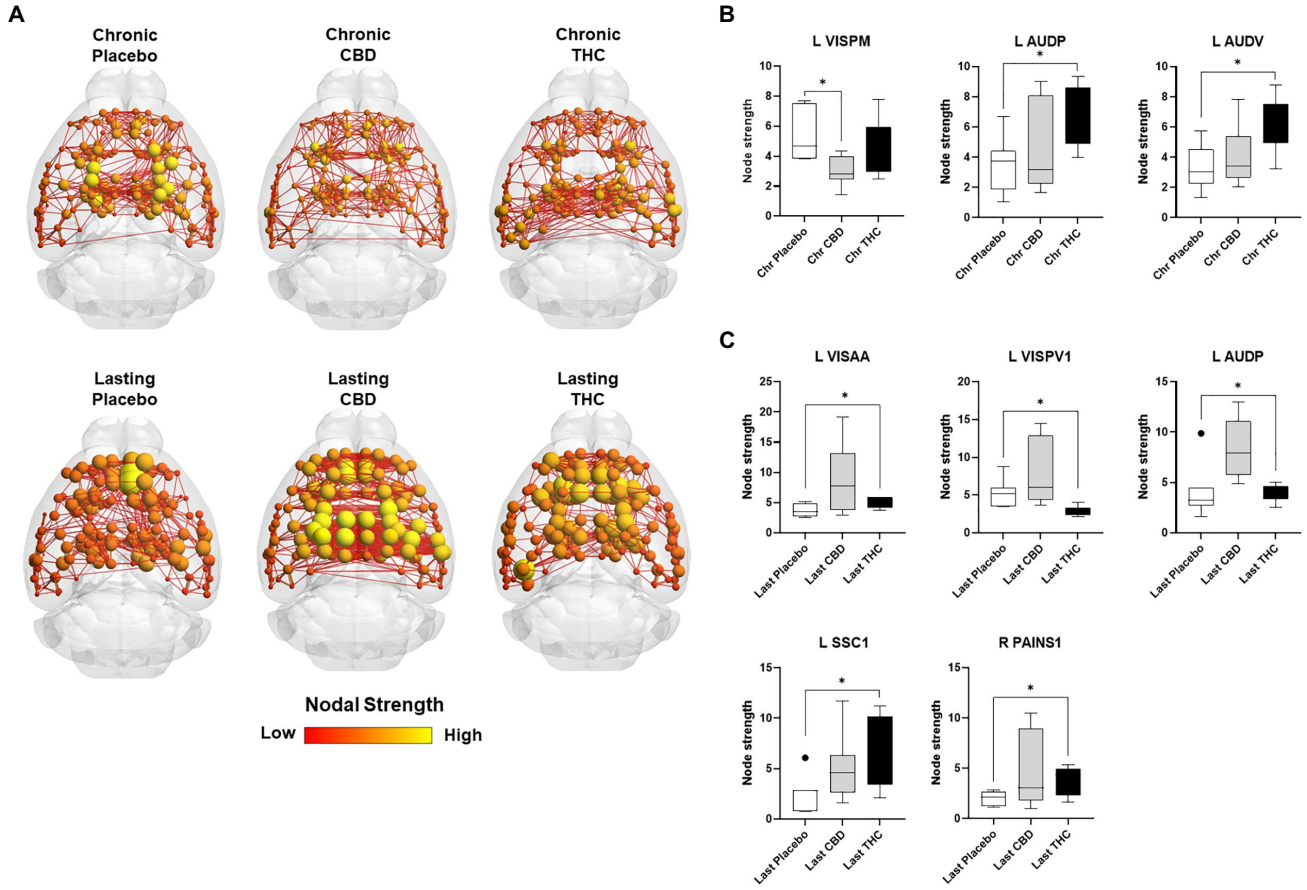
Regional differences in node strength. Regional differences in node strength following chronic placebo, CBD, or Δ9-THC over 28 days (chronic) and after a 2-week washout period (lasting). **(A)** Connectome maps of a mouse functional network highlighting mean node strength across groups. Spheres represent scaled area under the curve (AUC) values for node strength calculated over several edge density thresholds (2–40%). Size and color intensity of spheres reflect high-to-low values (see scale bar). Lines between nodes represent pairwise connectivity between nodes (these are shown at edge density of 6% for visual clarity). **(B)** Node strength values for chronic treatment mice (calculated at a 16% edge density threshold). **(C)** Node strength values for post-washout period (calculated at a 16% edge density threshold). Data are shown as mean ± standard error (*t*-test, FDR q = 0.05, *p* < 0.01). Abbreviations: L VISPM, left posteromedial visual cortex; L AUDP, left posterior auditory cortex; L AUDV, left ventral auditory cortex; L VISAA, left anterior area of the visual cortex; L VISPV1, left ventroposterior visual cortex node 1; L SCC1, primary somatosensory cortex; R PAINS1, right posterior agranular insula node 1.

**Figure 10:**
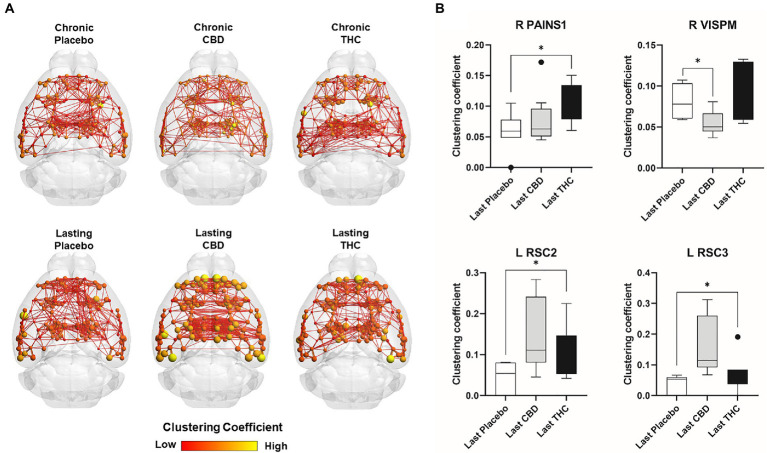
Regional differences in clustering. Regional differences in clustering coefficient following chronic placebo, CBD, or Δ9-THC over 28 days (chronic) and after a 2-week washout period (lasting). **(A)** Connectome maps of a mouse functional network highlighting mean clustering coefficient across groups. Spheres represent scaled area under the curve (AUC) values for clustering coefficient calculated over several edge density thresholds (2–40%). Size and color intensity of spheres reflect high-to-low values (see scale bar). Lines between nodes represent pairwise connectivity between nodes (these are shown at edge density of 6% for visual clarity). **(B)** Clustering coefficient values for chronic treatment mice (calculated at a 16% edge density threshold). Clustering coefficient values for post-washout period (calculated at a 16% edge density threshold). Clustering coefficient values for post-washout period (calculated at a 16% edge density threshold). Data are shown as mean ± standard error (two-way ANOVA with repeated measures, post-hoc t-tests with FDR q = 0.05, *p* < 0.05). Abbreviations: R PAINS1, right posterior agranular insula node 1; R VISPM, right posteromedial visual cortex; L RSC2/3, Left retrosplenial cortex nodes 2 and 3.

Figures 7, 8 show results for global functional network metrics. Figure 7 shows global network metrics as a function of various connection (edge) density thresholds and in Figure 8 we used an area under the curve (AUC) approach to generate a summary value for statistical analysis. Globally, functional networks differed as a function of imaging session (chronic vs. lasting, i.e., washout) and not with drug treatment. This is observed in AUC data in Figure 8 for network strength (F_1,37_ = 5.6, *p* = 0.02), characteristic path length (F_1,37_ = 5.8, *p* = 0.02), efficiency (F_1,37_ = 5.6, *p* = 0.02), and transitivity (F_1,37_ = 6.3, *p* = 0.02), but not assortative mixing and modularity. Modularity was greater in the lasting/CBD group than in lasting placebo (Dunnett’s test, *p* = 0.04, placebo vs. CBD). However, no effect was observed for Δ9-THC in this group (Figure 8). Figures 9, 10 show regional analysis. A two-way ANOVA with repeated measures was used to compare node strength across 148 nodes between control, CBD and Δ9-THC treated mice, within each session. False discovery rate correction was applied as implemented in Graphpad 9 (a two-stage step-up method). In Figure 9, the 3D connectome maps highlight robust differences in average node strength between lasting, i.e., washout versus chronic sessions after placebo, CBD, and Δ9-THC administration. Significantly reduced node strength following chronic CBD administration was observed in the left visual cortex (Figures 9B, L VISPM, placebo vs. CBD, *p* = 0.02) and increases in strength were observed in chronic Δ9-THC treated mice versus controls in subregions of the auditory cortex (Figures 9B, L AUDP and L AUDV, placebo vs. Δ9-THC, *p* = 0.03 and 0.2, respectively). In mice imaged after a 2-week washout period we observed increases in node strength in visual cortical subregions (L VISAA and LVISP1, placebo vs. Δ9-THC, *p* = 0.04 and 0.03, respectively), auditory cortex (L AUDP, placebo vs. CBD, *p* = 0.02), somatosensory cortex (L SSC1, placebo vs. CBD, *p* = 0.048) and posterior agranular insular cortex (R PAINS1, placebo vs. Δ9-THC, *p* = 0.02). No effect of CBD was observed, although the effect of this cannabinoid trended non-significantly in the same direction of Δ9-THC (Figure 9C). Figure 10 shows regional results for clustering coefficient per node. Similar to node strength the 3D functional connectome maps highlight mean clustering values differ between chronic and lasting imaging sessions (Figure 10A). However, there were regions with increased clustering coefficient values following 2-week washout after chronic Δ9-THC administration. These included the right posterior agranular insula (R PAINS1, placebo vs. Δ9-THC, *p* = 0.04) and the left retrosplenial cortex (L RSC2 and RSC3, placebo vs. Δ9-THC, *p* = 0.046 and0.02, respectively). The right visual cortex had lower node strength in CBD versus controls (R VISPM, placebo vs. CBD, *p* = 0.02; Figure 10B).

## Discussion

There is compelling evidence in preclinical and clinical studies that chronic use of cannabis during adolescence and early adulthood can have detrimental effects on cognitive function and brain structure (Ritchay et al., 2021). In contrast, translational research using healthy-aging, 18–24-month-old mice shows improved cognitive function to very low dose Δ9-THC (Bilkei-Gorzo et al., 2017; Sarne et al., 2018). There are also preliminary findings in humans that exposure to cannabis in old age may enhance learning and memory and quality of life (Calabrese and Rubio-Casillas, 2018). To study this topic, old mice (~20 months average) were exposed to daily treatments of vaporized placebo or cannabis high in CBD or Δ9-THC for 28 days. The plasma levels of THC and CBD achieved by inhaling vaporized cannabis were comparable to what has been reported in the human literature making the findings neurobiologically relevant. Changes in brain structure and function with multimodal MRI and behavioral tests for cognition, anxiety, and analgesia were performed. Imaging and behavior were recorded during chronic cannabis exposure and washout, i.e., 2 weeks after the cessation of drug exposure. Blood levels for Δ9-THC and CBD were comparable to those reported in human studies. There were unexpected changes in brain volume and gray matter microarchitecture around the midbrain dopaminergic system with Δ9-THC, altered connectomics with CBD and the absence of tolerance to the antinociceptive effects of chronic Δ9-THC exposure in old mice. Below, we discuss these findings with respect to the neuroadaptive and behavioral changes reported in preclinical and clinical studies on adolescent and adult subjects.

### Behavior

#### Tail Flick

There are numerous preclinical studies reporting that Δ9-THC, injected or inhaled, has antinociceptive effects. For example, Lichtman et al., found that inhalation exposure to cannabis produced antinociceptive effects that are both dependent on concentration and exposure time (Lichtman et al., 2000, 2001). There are differences across different age cohorts when comparing the antinociceptive potency and efficacy of Δ9-THC (1.0–18 mg/kg) on the tail withdrawal test (Craft et al., 2019). Significantly greater antinociceptive effects were observed in middle-aged rats (291–325 days post-natal) after being exposed to Δ9-THC compared to young adults (60–70 days), and significantly less antinociceptive effects were observed in adolescents (35–40 days) compared to young adults (Craft et al., 2019).

Although the acute effect of vaporized cannabis high in Δ9-THC was not tested, analgesia was tested in the tail flick assay after daily inhalation treatments over 28 days. Within 1 h of the last inhalation, exposed mice were tested for tail flick and found that mice exposed to inhaled Δ9-THC kept their tail in contact with the hot plate for a significantly larger amount of time (seconds) compared to the placebo and CBD groups, showing Δ9-THC has an antinociceptive effect. These results were surprising because there was no tolerance to chronic Δ9-THC exposure as compared to placebo. Tolerance has been reported in several behavioral tests including rat activity and the tail withdrawal test (Fried, 1976; Greene et al., 2018). Fried found that while the inhalation of cannabis decreased activity in rats, a tolerance is developed to this behavior and there is a cross tolerance that is observed between the cannabis smoke and IP injections of Δ9-THC (Fried, 1976). Gender differences were also reported in a study by Nguyen and colleagues in which repeated daily doses of Δ9-THC led to the development of a tolerance to the antinociceptive effects of Δ9-THC in females rather than males (Nguyen et al., 2018). However, we did not observe this in our study using primarily females.

The implications with respect to the human condition and quality of life for the elderly are significant. Chronic pain is very common in the elderly and is associated with significant morbidities such as limited mobility, social isolation, and a depressed mood (Schwan et al., 2019). Patients with acute, cancer-related, neurologic, or end-of-life pain are commonly prescribed pain medication like opioids due to their analgesic effects; however, because individuals have different levels of tolerance there is ample room for drug liability (Preuss et al., 2022). Additionally, patients can vary in their behavioral, cultural, emotional, and psychologic responses to pain which can make it challenging for health professionals to discern the difference between substance use disorder and reactions to pain (Preuss et al., 2022). Therefore, if cannabis is able to provide an analgesic effect without the patient requiring greater doses each time, it could be a viable treatment for chronic pain in the elderly (Mucke et al., 2018).

#### Open field test

Some of the earliest preclinical work on injected and smoked Δ9-THC showed changes in mobility and anxiety that could be assessed in the open field test. Bruijnzeel and colleagues investigated the effect of cannabis smoke exposure on locomotor activity, rearing, and anxiety-like behavior in rats (Bruijnzeel et al., 2016). Exposure to cannabis smoke containing 5.7% Δ9-THC, causes a brief increase in locomotor activity that is immediately followed by a prolonged decrease in the open field test. Rats exposed to inhaled cannabis smoke also spent more time in the center zone of the open field which indicates a decrease in anxiety-like behavior. This is consistent with findings that exposure to cannabis results in mild anxiolytic effects and an increase in self grooming behavior (Rusznak et al., 2018). Differences in Δ9-THC’s locomotor-suppressing effects were also indicated across different age groups with the greatest effect observed in young female rats (Craft et al., 2019). A comparison of methods for Δ9-THC administration, including orally, injected, and inhaled showed that all three routes lead to a significant reduction in open field activity with injection being the most effective (Fried and Nieman, 1973). However, conflicting results were reported by Lichtman who found no significant decrease in locomotor activity after inhalation exposure to Δ9-THC (Lichtman et al., 2000). This could perhaps be explained by results that suggest that exposure to smoke alone had pharmacological consequences (Lichtman et al., 2001).

The data collected here on old mice did not corroborate many of these findings. We found that after acute exposure to vaporized cannabis, there is a significant difference between the test groups in the percent of the time that the mice spent in the center of the field which is presumably a measure of anxiety. However, after chronic exposure to cannabis, there was no significant difference between groups in the percent of the time in the center of the field, suggesting tolerance. This surrogate measure of anxiety is reported to wane with repeated treatments with Δ9-THC, particularly at higher doses (Sharpe et al., 2020). It should be noted that although there was only a significant difference in the distance traveled (cm) at chronic exposure timepoint between high Δ9-THC and placebo, mice receiving high Δ9-THC consistently traveled longer on average than the other two groups no matter the timepoint.

#### Novel object recognition

Much of the clinical work on cannabis use in adolescence and middle adults has warned of the detrimental effects on learning and memory (Dahlgren et al., 2016). Heavy cannabis use, especially in adolescence, had been associated with adverse effects on several systems including executive, emotional, reward, and memory processing which increase the risk of mental illnesses including addiction and psychosis (Bloomfield et al., 2019). Calabrese and Rubio reported cannabis use in old mice enhanced cognitive function, but the effects were dose-dependent, with only chronic low doses improving neurological function (Calabrese and Rubio-Casillas, 2018). Likewise, a study conducted on mice aged 12 to 18 months found that extremely low doses of Δ9-THC administered continuously through an osmotic minipump for several weeks reversed age-related decline in cognitive performance including memory, learning, and flexibility; and after treatment, closely resembled Δ9-THC-free animals aged 2 months (Bilkei-Gorzo et al., 2017). This suggests that exposure to cannabis restores the CB1 signaling in the elderly and could be an effective strategy to treat age related cognitive decline (Bilkei-Gorzo et al., 2017). Here we found that mice acutely exposed to cannabis high in Δ9-THC led to a decrease in the investigation ratio, suggesting that exposure to high Δ9-THC may not increase cognitive functioning. There were no significant differences between test groups during or after chronic exposure, suggesting tolerance. Indeed, much of the preclinical and clinical literature reports little or no positive effects of cannabis on learning and memory, and most report only negative effects. Aso and colleagues report that while natural cannabinoids may still be effective in reducing memory impairment in a mouse model of Alzheimer’s disease, it was not effective in modifying the possession of or reducing the glial reactivity associated with the early stages of the disease(Aso et al., 2016). Moreover, results indicate that cannabis had no effect on cognitive impairment in healthy aging wild-type mice.

#### Drug liability

There is clear evidence from preclinical studies that exposure to Δ9-THC either injected or inhaled can have neuroadaptive changes in reward circuitry resulting in symptoms of withdrawal in the absence of cannabis or treatment with CB1 receptor antagonists. Acutely, cannabis and Δ9-THC produced a range of effects on the glutamatergic, GABAergic, and dopaminergic systems (Bloomfield et al., 2019). The protective effects on the cognitive function of Δ9-THC can be blocked by the CB1 receptor antagonist and mimicked by the CB1 agonist ACEA (Fishbein-Kaminietsky et al., 2014). The reinforcing effect of some synthetic CB1 agonists has been reported in both rats and mice which is consistent with previous studies that concluded Δ9-THC has a significant abuse liability compared to other drugs (Justinova et al., 2005). Although drug liability was not tested, there was a pronounced change in the volume of brain areas comprising the dopaminergic system. The FA values for brain areas that make up the dopaminergic system such as accumbens shell, accumbens core, ventral pallidum and the white matter tracts are lower, suggesting disorganization. Would these changes result in altered responsivity to repeated cannabis exposure (i.e., tolerance) and be foundational in any addictive behavior? This is consistent with research that indicates that the dopaminergic system plays a key role in addiction and can be targeted to treat patients with addiction (Solinas et al., 2019). The change in the volume of the dopaminergic system that is observed in our results could indicate that there is a likelihood of addiction after repeated use of cannabis.

### Neuroimaging

#### Voxel based morphometry

Psychiatric and neurological conditions are commonly characterized by changes in brain structure using VBM. This imaging modality is particularly relevant in longitudinal studies following brain development in normal aging and in response to drugs. Of particular interest in this field are morphological changes in the brain following chronic use of cannabis. Medina and colleagues found that adolescent chronic cannabis users developed significantly larger vermis volumes than their control counterparts(Medina et al., 2010). Another study found that the age of onset of cannabis use influenced white matter coherence and accumbens shape (Orr et al., 2016). A common finding is a general reduction in brain volume as age increases. This poses a pertinent question: Does the human brain get smaller because of aging, or because of environmental influences on brain size? Studies have reported significant reductions in brain size after the age of 40 (Svennerholm et al., 1997). The culprit of this shrinkage is less clear. Some studies report age related reductions in grey matter are linked to neuronal atrophy, though this is not agreed upon (Kolb and Whishaw, 1998; Trollor and Valenzuela, 2001). The underlying cause of brain shrinkage is an important consideration when discussing the effects of cannabis on the brain.

While this was not a within-subject design following changes in brain volume in each mouse before and after 28 days of inhaled cannabis we were able to make comparisons between mice for each experimental condition, e.g., placebo, CBD, and Δ9-THC at the cessation of cannabis exposure. While there were few if any significant changes comparing all 139 brain areas, we noted that all areas comprising the DA system were smaller in volume in the Δ9-THC groups. In this study, we witnessed the aforementioned reduction in brain size with age; however, it took place in the DA system of Δ9-THC group mice, indicating that drug administration played a role in brain shrinkage. There are other studies finding that Δ9-THC influences brain volume; however, those findings are inconsistent and often report increases in brain volume. One study found that heavy cannabis users had larger anterior cerebellar volumes (Cousijn et al., 2012). Another study reported an increase in basal ganglia structures (Moreno-Alcazar et al., 2018). To our knowledge, our findings are the first reporting a significant decrease in the volume of the dopaminergic system following chronic Δ9-THC administration.

This sensitivity of the midbrain DA system to chronic cannabis exposure was made more intriguing following the 2-week washout a time lapse that should have effectively cleared brain and plasma of THC (Huestis, 2005). In this short period of time, the volumes in these same brain areas reversed presenting as significantly greater than placebo. This finding is supported by our DWI findings, which report a decrease in FA following the two-week washout. Why of all of the different brain regions was the midbrain DA system most sensitive to THC? DA neurons in the midbrain are sensitive to their own metabolism generating reactive oxygen species that creates an environment of oxidative stress and neuroinflammation (Hirsch and Hunot, 2009; Meiser et al., 2013). DA neurons become more vulnerable with aging, a risk factor for Parkinson’s disease (Skaper et al., 2018). While not confirmed by histochemistry, it is possible the midbrain DA system might present with some level of underlying neuroinflammation. The changes in volume may reflect changes in cell swelling from reduced inflammation as a consequence of THC (Burstein, 2015; Mori et al., 2017; Tangestani Fard and Stough, 2019). A more plausible explanation is the change in osmolarity that accompanies neuronal activity and astrocytic swelling that is rapidly corrected by AQP4 convection of water across the perivascular space (Cai et al., 2021; Ferris, 2021).

#### Diffusion weighted imaging

This dramatic change in morphology with a two-week washout was also seen with DWI. There were no significant differences in FA or ADC for any experimental group, during 28 days of Δ9-THC administration. However, after only two-weeks of washout, there were significant changes in FA values but not ADC. These significant changes in FA were significant reductions, indicating a decrease in the directionality of water. The lower FA would suggest reduced gray matter organization or expanded gray matter volume. While there is evidence of rapid changes in brain morphology, e.g., decrease in dendritic spines, perineuronal nets, capillary density, and extracellular matrix, this robust change in anisotropy across so many brain areas would seem unprecedented. It is more likely that homeostatic regulation of the fluid balance between the intracellular and extracellular compartments is a more feasible explanation for the decrease in FA. These decreases occurred in a variety of areas, notably in the DA system. This finding is consistent with the VBM findings, indicating that a decrease in water volume following the 14-day washout resulted in both increases in the volume of the areas and decreased directionality of the water inside neurons and/or glia.

#### Connectomics

The lack of robust global changes in functional connectomic measures in response to chronic Δ9-THC or CBD administration in aged mice is somewhat surprising considering the extensive behavioral and central effects observed at earlier adolescent ages reported across many rodent studies (Bruijnzeel and Tandon, 2018; Bara et al., 2021). In aged mice, we observed that after a 2-week washout period from chronic CBD exposure (lasting CBD) there was an increase in modularity relative to placebo. Functional connectivity networks have been previously reported to follow a modular organization in humans and other species including rodents (Bertolero et al., 2018; Liang et al., 2018). The interspecies persistence of this network property could reflect its importance to functional specialization (through segregation of activity) of groups of nodes (Bertolero et al., 2015) and may serve as an indicator of functional recovery after CNS damage (Siegel et al., 2018). Reductions in modular organization have been associated with increased global integration of functional connectivity (Daws et al., 2022). Increases in modularity are associated with increased cognitive performance on a range of tasks (Bertolero et al., 2015, 2018). Conversely, reductions in modularity (increased global integration) have been reported in response to psilocybin treatment and reduced symptoms of depression (Daws et al., 2022). The lasting effects of CBD seem consistent with the former findings and not the latter. CBD could increase modularity in functional connectivity networks, and this associates with cognitive performance, a relationship that warrants investigation using combined fMRI and behavioral analysis.

Our node-based analysis indicated that there were differences in node strength between Δ9-THC, CBD, and the placebo control group, and the specific regions affected varied as a function of the delay since last treatment (chronic vs. lasting effects). The regions affected by either Δ9-THC or CBD play a role in a range of sensory cortical functions such as, visual, auditory, interoception, somatosensorial. Differences in connection strength in the observed cortical areas could be linked to strengthening of existing connections (plasticity) or increases in the total number of connections to these regions (increased divergence of connections). The effects of endogenous cannabinoid receptors on synaptic plasticity *via* changes in pre-synaptic strengthening are well established and can encompass excitatory synapses in these regions (Winters and Vaughan, 2021). Similar mechanism might contribute to changes in clustering coefficient observed in a subset of these cortical areas.

There have been few studies that have investigated the effect of cannabinoid administration on functional connectivity derived graph theory metrics. All the studies to date have been in human subjects but none have studied the effects of Δ9-THC or CBD in aged subjects. General hyperconnectivity in default mode and dorsal and ventral attention networks was identified in chronic cannabis smokers relative to occasional users (Ramaekers et al., 2022). Local efficiency and global measures of connectome strength and transitivity were greater in 18–55-year-old cannabis users compared to matched controls (Hall et al., 2021). Effects of Δ9-THC on local and global efficiency have also been reported in 27–40 year old subjects with neuropathic pain and this was correlated with analgesic effects of the drug (Weizman et al., 2018), Differences in clustering and efficiency in functional networks have been reported in epileptic patients with and without CBD treatment (Morales Chacon et al., 2021). Although these studies indicate consistent effects of cannabinoid treatment on functional connectome measures of integration (clustering coefficient, transitivity, strength) and efficiency, none investigated the effects of Δ9-THC and CBD in the aged. Further studies are needed, particularly in animal models of aging in which there is optimal experimenter control of drug treatment regimen and better isolation of polydrug versus single drug use.

Why did CBD influence connectivity and not THC? Central receptor targets for the effects of CBD have been described. These include GRP55, TRPV1, CB2 (Perez et al., 2022), and some recent work has identified actions *via* either 5HT1A or CB1 in hippocampus (Franzen et al., 2023). Due to the lack of similar lasting effects of THC on network strength compared to CBD, a CB1 mediated action might be unlikely to mediate the observed differences. The effects mediated via serotonergic 5HT1A receptors in hippocampus are associated with fear learning and could be an interesting target to investigate in future studies using fMRI and fear conditioning paradigms. However, as indicated above, there are several other interesting targets of CBD, including those involving neuronal-glial-immune interactions, which can also produce persistent effects.

### Limitations

There are some limitations to this study. First, the study was conducted primarily on female animals; and therefore, potential gender differences could not reliably be assessed. Ideally, the study would have been conducted on a sample of both males and females so it would be more representative of human elderly populations. However, the mice were generously gifted from Delaware; and due to the rarity of elderly test animals, having an equal distribution was not possible in this circumstance. Another limitation is that the select measure of locomotor activity, rearing, and anxiety-like behavior and cognition was the open field test and novel object preference, respectively. Although both are valid measures supported by literature in the field, there are several other, more complex tests that could have revealed more about the effects of cannabis on the elderly. Another concern with the behavioral studies was the repeated testing over time and whether adaptation was affecting the results. The altered behaviors could also be explained by changes in CB1 and CB2 binding. Have the ADME properties and binding affinities for THC and CBD changed with age and/or chronic exposure and washout? The data from the study indicate that the animals did not develop a tolerance to the antinociceptive effects of cannabis; however, whether this was true for drug liability was not tested. Therefore, should the study be repeated, it may be worthwhile to expose the subjects to rimonabant a CB1 antagonist to determine if withdrawal symptoms were present. Lastly, immunohistochemistry would be beneficial to definitively conclude if the underlying mechanism is a neuroinflammatory response and/or alterations in grey matter architecture.

### Summary

Results of the study indicate that cannabis may have several surprising effects on the aging brain. Cannabis had antinociceptive effects in the elderly mice without tolerance, suggesting cannabis may be an effective treatment for chronic pain in the elderly. However, this study did not find that chronic exposure to vaporized cannabis had any significant effects on anxiety or cognition. Imaging showed a decrease in brain volume of the dopaminergic system as well as significant changes in FA values which indicated a reduction in gray matter volume. Cannabis high in CBD but not Δ9-THC increased network strength and efficiency, an effect that persisted after washout. More research must be conducted to understand the mechanism behind these effects.

## Data availability statement

The original contributions presented in the study are included in the article/Supplementary material, further inquiries can be directed to the corresponding author.

## Ethics statement

The animal study was reviewed and approved by Northeastern University IACUC.

## Author contributions

AS, CF, PK, HB, and MF: concept, drafting, and interpretation. AS, FC, MG, RO, MF, and CJ: execution and analysis. All authors contributed to the article and approved the submitted version.

## Funding

MF is supported by NIH grants R01AG070913, R21AG065819, and UH3NS106938; HB by DA041208; and MG by NIH-NIGMS Centers of Biomedical Research Excellence (COBRE): 5P20GM103653.

## Conflict of interest

CF has a financial interest in Animal Imaging Research, the company that makes the radiofrequency electronics and holders for awake animal imaging. CF and PK have a partnership interest in Ekam Solutions the company that develops 3D MRI atlases for animal research.

The remaining authors declare that the research was conducted in the absence of any commercial or financial relationships that could be construed as a potential conflict of interest.

## Publisher’s note

All claims expressed in this article are solely those of the authors and do not necessarily represent those of their affiliated organizations, or those of the publisher, the editors and the reviewers. Any product that may be evaluated in this article, or claim that may be made by its manufacturer, is not guaranteed or endorsed by the publisher.

## Footnotes

^1^ https://www.cbica.upenn.edu/sbia/software/dramms/index.html

^2^ http://www.sop.inria.fr/asclepios/software/MedINRIA/index.php

^3^ http://afni.nimh.nih.gov/afni/

^4^ http://fsl.fmrib.ox.ac.uk/fsl/

^5^ https://www.cbica.upenn.edu/sbia/software/dramms/index.html

^6^ https://www.slicer.org/
